# Environmental impacts of restructuring the EU’s natural gas supply and consumption: Learnings from the 2022 energy crisis

**DOI:** 10.1016/j.isci.2024.111575

**Published:** 2024-12-12

**Authors:** Lucas F. Santos, Robert Istrate, Niall Mac Dowell, Gonzalo Guillén-Gosálbez

**Affiliations:** 1 Institute for Chemical and Bioengineering, Department of Chemistry and Applied Biosciences, ETH Zürich, Vladimir-Prelog-Weg 1, 8093 Zürich, Switzerland; 2 Department of Chemical Engineering, State University of Maringá, Avenida Colombo 5790, Maringá 87020900, Brazil; 3 Institute of Chemical Process Engineering, University of Alicante, Ap. Correos 99, 03080 Alicante, Spain; 4 Institute of Environmental Sciences (CML), Leiden University, Einsteinweg 2, 2333 CC Leiden, the Netherlands; 5 Centre for Environmental Policy, Imperial College London, Exhibition Road, London SW7 1NA, UK; 6 Centre for Process Systems Engineering, Imperial College London, Exhibition Road, London SW7 2AZ, UK

**Keywords:** Energy policy, Engineering, Energy systems

## Abstract

In 2022, the European Union put forward the REPowerEU plan in response to Russia’s invasion of Ukraine, aiming at enhancing short-term energy security by diversifying imports and reducing natural gas demand while accelerating the deployment of renewable alternatives in the long term. Here, we quantify the life cycle environmental impacts of both REPowerEU’s short-term measures, including the controversial extended coal-fired power plant operations, and how the first year of the crisis was managed in practice. We find that the policy measures’ impact on greenhouse gas (GHG) emissions would be negligible, although they could have detrimental effects on other environmental categories. In practice, GHG emissions dropped by 8.6% driven by energy savings, yet other environmental burdens worsened, primarily due to coal and oil use. Our results could support the development and analysis of long-term policies to enhance energy security via natural gas demand reduction while considering multiple environmental sustainability indicators to avoid collateral damage.

## Highlights

• REPowerEU (short-term) would reduce natural gas imports maintaining GHG emissions
• Energy savings in the natural gas crisis management cut GHG emissions by 8.6%
• Delaying the phase-out of coal worsens categories beyond climate change
• Optimized actions to cut natural gas demand can avoid environmental burden shifting

## Graphical abstract

**Figure:**
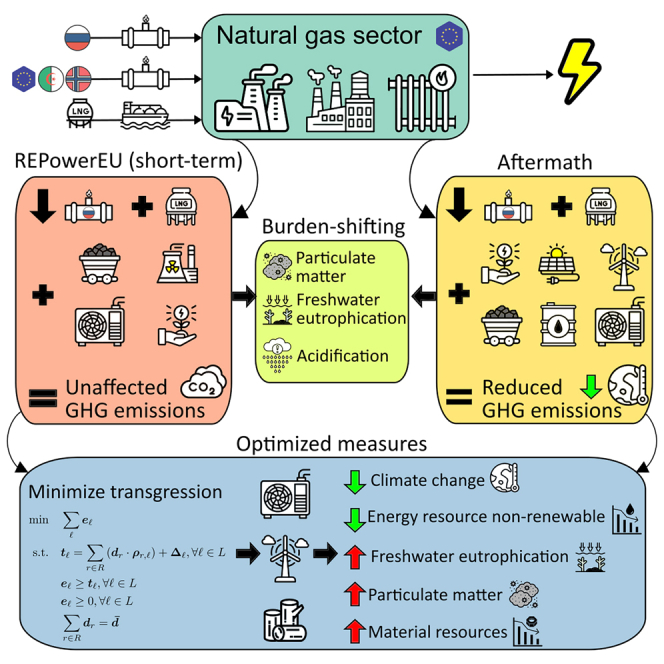

## Introduction

Russia’s invasion of Ukraine has led to a geopolitical crisis that prompted the European Union (EU) to reconsider its reliance on Russian oil, coal, and natural gas imports. The situation is particularly complex when it comes to natural gas since EU countries have historically strongly relied on Russian natural gas, i.e., approximately 38% of the EU’s consumption in 2021,^1^^,^^2^ making it hard to abandon the current status quo. Diversification of suppliers aimed at reducing Russian gas imports is further hampered by capacity constraints on piped natural gas and liquefied natural gas (LNG).^3^ Overall, the geopolitical conflict, post-pandemic economic recovery, strong reliance on fossil fuels, and mismatch between energy supply and demand have resulted in a temporary surge in energy prices^4^^,^^5^ and subsequent energy,^6^ food security,^7^ and poverty concerns, with European natural gas peak commodity prices as high as 240 euros (EUR) per megawatts hour (MWh) monthly average in August 2022 (vs. 38.5 EUR MWh^−1^ pre-invasion and 32.3 EUR MWh^−1^ as for the end of 2023).

Prompted by this context, in May 2022, the European Commission released the REPowerEU action plan to enable a total phase-out of Russian gas imports by 2027 based on three pillars, i.e., saving energy, diversifying supplies, and accelerating clean energy.^3^ In the short term, the plan targeted a quick 110 billion cubic meters (bcm) reduction of gas imports from Russia (ca. 75% of imports from Russia and ca. 29% of total imports in 2021^1^) by deploying a set of policy actions. These included diversifying pipeline imports, increasing LNG imports, saving energy, delaying the phase-out of coal and nuclear power plants, and electrifying heating.^8^ In the medium and long term, to fully phase out Russian gas imports, the REPowerEU plan proposes additionally deploying heat pumps and improving energy efficiency in buildings (37 bcm), expanding wind and solar power capacity (21 bcm), deploying sustainable bio-methane (17 bcm), electrifying heating and replacing natural gas in industries (12 bcm), and increasing renewable hydrogen production (27 bcm).^8^

Looking retrospectively at the first year of the 2022 energy crisis, the EU reduced natural gas demand by 55 bcm,^9^ representing the steepest drop in history. The leading factors driving this decrease included elevated gas prices, a mild winter, and prompt policy responses to the crises. These drivers all together led to changes in behavior and fuel substitution among households, fuel switching and reduced electricity consumption in the power sector, and production curtailment and fuel switching within the industrial sector.^9^

The REPowerEU plan and the actual management of the energy crisis demonstrated the feasibility of restructuring the EU’s energy system to reduce reliance on natural gas. Given that the priority goal of this restructuring was to enhance energy security during the crisis period, previous works have thoroughly discussed the associated techno-economic challenges.^10^^,^^11^^,^^12^^,^^13^^,^^14^^,^^15^^,^^16^^,^^17^^,^^18^ Additionally, effort has been made to assess the environmental impacts of the 2022 natural gas crisis in the electricity generation mix of Italy based on government plans and modeling scenarios.^19^ However, the broad environmental implications of the natural gas crisis, including the impact on the EU’s greenhouse gas (GHG) emissions, remain largely unexplored, despite encompassing actions potentially affecting the environment in distinct ways. Specifically, considering the proposed short-term measures in the REPowerEU plan, energy savings and delaying the phase-out of nuclear plants could help curb GHG emissions. Additionally, the phase-out of Russian gas could mitigate methane leakages.^20^ In contrast, a temporary turn back to coal in power generation would raise GHG emissions,^21^^,^^22^ while likely exacerbating other environmental and human health impacts due to coal mining and combustion (e.g., toxic airborne emissions^23^^,^^24^). Increasing LNG imports could raise energy consumption across the supply chain owing to the energy required to liquefy the natural gas^25^^,^^26^ while also leading to higher prices and energy security issues due to the sudden rise in global demand, potentially affecting other important non-EU LNG importers (e.g., Japan, South Korea, Bangladesh, and Pakistan).^27^ Moreover, the potential benefits of electrifying heating will depend on the composition of regional power mixes.^28^ Since natural gas consumption is a major driver of the EU’s GHG emissions (ca. 16% of total energy-related CO_2_ emissions in 2021^29^), expanding our limited knowledge of the environmental impacts of restructuring supply and consumption is deemed essential. Moreover, understanding the impacts of the REPowerEU plan compared with the actual crisis management could provide valuable insights for policy-making, ultimately supporting more sustainable medium- and long-term planning.

Here, we assess the environmental impacts of restructuring the EU’s natural gas supply and consumption to cope with the energy crisis resulting from Russia’s invasion of Ukraine. More specifically, we evaluate the short-term policy actions of the REPowerEU plan to quickly reduce 110 bcm of Russian gas in the first years of the crisis. In an ex-post analysis, we also assess the extent to which the short-term measures were realized and evaluate the environmental implications of the actual crisis management in the same period. Applying life cycle assessment (LCA), we find that the short-term measures in the REPowerEU plan would hardly affect current GHG emissions levels because increased GHG emissions from temporally turning back to coal could overshadow the reductions in emissions from other measures. Yet, a delayed phase-out of coal-fired power plants would worsen other impact categories, namely acidification, eutrophication, ionizing radiation, land use, particulate matter formation, and water use. An 8.6% GHG emission drop was identified in the first-year aftermath mainly driven by energy savings. Nevertheless, impacts on acidification, eutrophication (freshwater, marine, and terrestrial), ionizing radiation, land use, particulate matter formation and photochemical oxidant formation could worsen by as much as 66% compared to pre-invasion levels, primarily due to the increased coal and oil use. Overall, our results shed light on the environmental implications of the natural gas crisis management in the EU countries from a policy and aftermath viewpoint. Our study could support the development and analysis of long-term strategies to further reduce the natural gas demand to enhance energy security in the context of geopolitical instability^21^^,^^22^^,^^30^ and pursue climate and sustainable development goals.^31^^,^^32^

## Results

### EU’s natural gas supply and consumption scenarios before and after the 2022 crisis

We defined three scenarios to study the EU’s natural gas supply and consumption for energy purposes (electricity production and heating in industry, households, and service sector) that describe the pre-invasion (Base), crisis management based on proposed short-term policies (REPowerEU), and the first year of managing the crisis (Aftermath). We omitted natural gas used for non-energy purposes, e.g., as feedstock for chemical production (mostly ammonia and methanol synthesis), as this demand represents only around 4% of the whole EU’s natural gas demand.^2^ Moreover, in contrast to other uses of natural gas particularly for power and heat generation, such demand would be much harder to replace due to the lack of sufficiently matured alternative chemical technologies and associated infrastructure.^33^^,^^34^^,^^35^ For comparison purposes, the three scenarios are designed to meet the same electricity and heating demand. This means that, for example, if coal-fired and nuclear power plants are considered to replace natural gas, they must match the amount of electricity generated by the natural gas-fired power plants.

**Figure 1:**
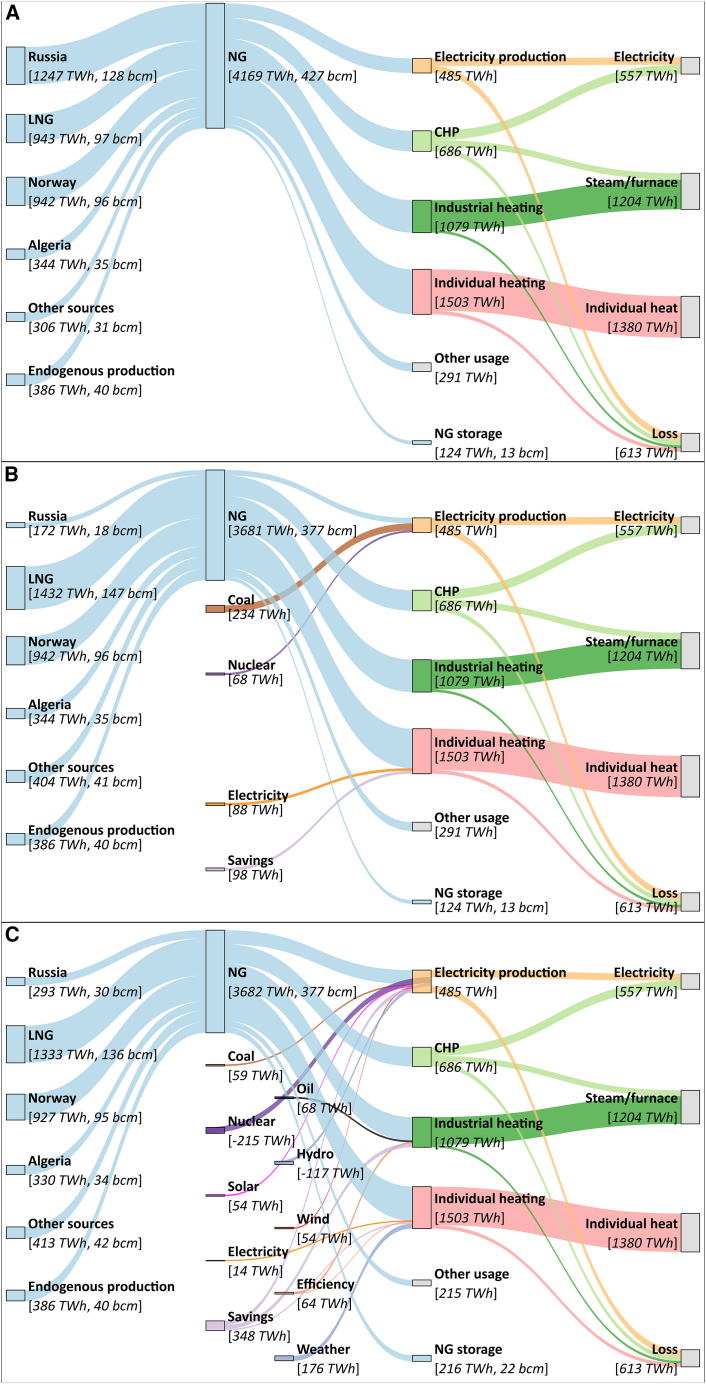
Overview of the assessed scenarios of EU’s natural gas supply and consumption for electricity production and heating (A) Base scenario shows the situation in the twelve months from June 2021 until the end of May 2022. Here, the total natural gas consumption is 427 bcm, out of which 384 bcm is used to supply 557 TWh of electricity, 213 TWh of heat from CHP, 991 TWh of heat in industry, and 1,380 TWh of heat in households. Russia supplies about 40% of the natural gas, accounting for 128 bcm by pipelines and the other 16 bcm as LNG. (B) REPowerEU scenario depicts the hypothetical deployment of the short-term policy actions outlined in the REPowerEU plan to reduce the imports of Russian gas by 110 bcm considering the twelve months from June 2022 as representative year. These measures include 50 bcm more of LNG from the U.S., 10 bcm of natural gas from other sources (i.e., the Southern Corridor), 10 bcm from savings in households, 9 bcm from electrifying heating in households, 24 bcm from delaying the phase-out of existing coal-fired power plants, and 7 bcm from abandoning the phase-out of existing nuclear power plants. Here, natural gas consumption would drop to 334 bcm while satisfying the same electricity and heating demand as in the Base scenario. (C) Aftermath scenario comprises the first year of managing the crisis in practice considering the twelve months from June 2022 as the representative year. Here, a 98 bcm reduction in Russian gas imports was realized while LNG and other sources of piped gas increased by 39 bcm and 11 bcm, respectively. On the demand side, the reduction of 50 bcm of natural gas, 215 TWh in nuclear power, and 117 TWh of hydropower were compensated by increased coal (59 TWh), oil (68 TWh), solar power (54 TWh), wind power (54 TWh), heat pumps (14 TWh), higher efficiencies (64 TWh), savings (348 TWh), and mild weather (176 TWh) Notice that negative flows from electricity production indicate an increased demand from the natural gas sector to cover for reduced nuclear and hydropower capacities. CHP: combined heat and power. LNG: liquefied natural gas. NG: natural gas. See also Tables S1–S3.

The Base scenario comprises the pre-invasion of Ukraine conditions, assuming the twelve months from June 2021 to the end of May 2022 (when the REPowerEU plan was launched) as the representative year (Figure 1A). Here, the EU consumed 427 bcm of natural gas, of which 387 bcm were imported^1^ and 40 bcm supplied internally.^36^ Leaving aside other natural gas applications (291 TWh, mostly for non-energy industrial feedstock) and gas storage stock change year-over-year (13 bcm to storage),^1^ the remaining ca. 384 bcm of natural gas was consumed in the EU annually for heating in households (40% of the total), heating in the industry (29%), combined heat and power (CHP) generation (18%), and electricity production (13%).^2^ This natural gas was used to supply about 557 TWh of electricity, 213 TWh of heat from CHP, 991 TWh of heat in industry, and 1,380 TWh of heat in households, with losses due to energy efficiencies accounting for 613 TWh. Russia supplied about 40% of the consumed natural gas for energy use, representing 144 bcm year^−1^ (128 bcm by pipelines and 16 bcm as LNG). The remaining gas was supplied by Norway (96 bcm by pipelines); Algeria (35 bcm by pipelines and 9 bcm of LNG); the U.S. (39 bcm of LNG); Qatar (17 bcm of LNG); Nigeria (16 bcm of LNG); and other sources, including the UK, Azerbaijan, and Libya. Further information regarding natural gas supply and energy demand in the EU countries can be found in Tables S1–S3 in the Supplemental Information (SI).

The REPowerEU scenario depicts a situation where the short-term policy actions outlined in the REPowerEU plan would be deployed to reduce Russian gas imports by 110 bcm (Figure 1B). Here, we define the twelve months from June 2022 as the representative year of the natural gas crisis management, i.e., starting immediately after the plan was released and when the Russian import decrease intensified.^1^ Considering existing pipelines and regasification infrastructure, diversifying suppliers would be insufficient to compensate for the natural gas disruption.^11^ Specifically, the EU could accommodate 50 bcm more of LNG and 10 bcm of natural gas from the Southern Corridor via pipeline.^8^ Hence, this alternative new supply mix could overall provide 334 bcm of natural gas (vs. 384 bcm as before the crisis), resulting in a potential shortage of 50 bcm year^−1^, which should be addressed from a demand-side perspective, i.e., reducing the natural gas consumption. Short-term measures to accomplish such reduction following the REPowerEU plan^8^ include (1) 10 bcm from decreasing heating needs in households and the service sector by reducing 1°C thermostats’ set points; (2) 9 bcm from electrifying heating in households; (3) 24 bcm from delaying the phase-out and increasing the operating hours of existing coal-fired power plants, and (4) 7 bcm from delaying the phase-out of existing nuclear power plants. Notice that the short-term demand-side measures (1, 3, and 4) are regarded as temporary in the REPowerEU plan, and they are expected to be rolled back as the medium- and long-term policy actions are implemented by 2027.^8^ Note also that, for comparison purposes, the scenarios are designed to meet the same electricity and heating demand. This means that, for example, coal-fired and nuclear power plants have to match the amount of electricity generated by natural gas-fired power plants consuming 24 and 7 bcm of natural gas, respectively.

The Aftermath scenario considers the first year of managing the crisis in practice, based on the report from the International Energy Agency (IEA) detailing what drove the record fall in natural gas demand^9^ and employing the same representative year as in the REPowerEU scenarios (Figure 1C). Here, a big shift to LNG indeed took place in the supply mix of natural gas to EU countries, increasing 39 bcm for a total of 136 bcm. The reduction of Russian gas supply was realized up to 89% out of the forecasted 110 bcm reduction in the first year of the crisis, totaling 30 bcm of piped natural gas. As anticipated in the REPowerEU, diversifying pipe gas imports was responsible for providing an extra amount (11 bcm) of natural gas. Minor changes happened in Norway and Algeria imports as well as endogenous production as these were already at maximum capacities.^37^^,^^38^^,^^39^ In order to deal with the 50 bcm shortage in natural gas a range of actions took place. In the power sector, the demand for natural gas actually increased because of unexpected maintenance in nuclear power plants and record low hydropower production, reducing their respective production by 110 and 60 TWh.^9^ Overall, to guarantee the 247 TWh of electricity output from the EU’s natural gas-fired power plants and recover the nuclear and hydropower losses, more operating hours in coal-fired power plants (59 TWh), deployment of solar (54 TWh) and wind power (54 TWh), and electricity savings (147 TWh) played important roles. In the industrial sectors, 68 TWh of oil, 29 TWh of savings from increased efficiency, and 147 TWh of savings from industrial production curtailments helped reduce the demand for natural gas. Finally, the use of natural gas in households and the service sector to deliver 1,380 TWh of heating was partially compensated by heat pumps (14 TWh) and reduced need due to mild winter (176 TWh), increased thermal efficiencies (34 TWh), and human behavior-driven savings (55 TWh).

### Limited impact on GHG emissions from the EU’s natural gas short-term crisis management

**Figure 2:**
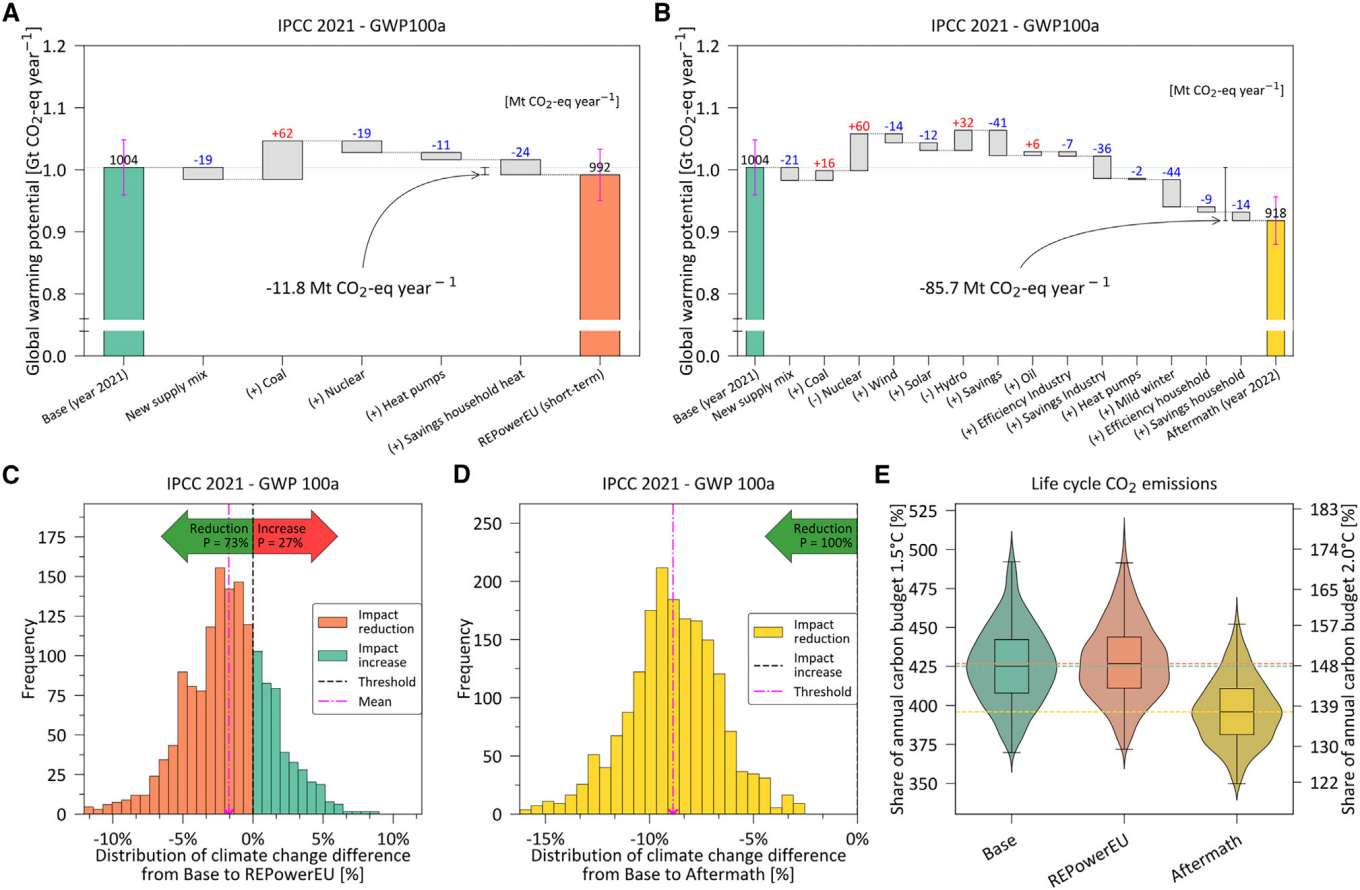
Climate change impacts of restructuring the EU’s natural gas supply and consumption (A) and (B) display, respectively, the climate change impacts of the REPowerEU plan and Aftermath scenarios considering the 100-year global warming potentials (GWPs). (C) and (D) present the distribution of the difference in climate change impacts from the Base to REPowerEU and Aftermath scenarios, respectively. Furthermore, it calculates the probability that the alternative scenario emits more GHGs than the Base scenario, denoted in percentage. (E) Shows the life cycle CO_2_ emissions distribution from 1000 Monte Carlo simulations for the three scenarios compared with the EU’s yearly carbon budget consistent with a high likelihood (67%) of limiting global warming to 1.5°C (left axis) and 2.0°C (right axis) above the pre-industrial level downscaled by population on an egalitarian basis (Table S17). All the scenarios heavily transgress the whole EU budget for the more ambitious temperature 1.5°C goal, and they slightly surpass the budget in the 2.0°C case. Error bars indicate the quartile coefficient of dispersion (Methods S1). See also Figures S1–S3, S5, S6, and S10 and Table S17.

Natural gas supply and consumption in the Base scenario emit 1.00 gigaton of CO_2_ equivalent per year (Gt CO_2_-eq year^−1^) over the whole life cycle from extraction, processing, and transport until its final use to generate electricity or heat (Figure 2C). The short-term policy actions in the REPowerEU plan would have a minor impact on the EU’s GHG emissions, despite the controversial delayed phase-out of coal-fired power plants. Here, a temporary 24 bcm switch from natural gas- to coal-fired power plants would increase emissions by 62 Mt CO_2_-eq year^−1^ with the other measures concurrently avoiding 73 Mt CO_2_-eq year^−1^, resulting in a net reduction of 12 Mt CO_2_-eq year^−1^ (a 1% reduction over the Base).

Saving 10 bcm of natural gas in households has the highest mitigation potential (24 Mt CO_2_-eq year^−1^), followed by compensating 7 bcm of natural gas for electricity with nuclear power plants (19 Mt CO_2_-eq year^−1^). Moreover, compensating 9 bcm of natural gas via heat pumps in households would avoid 11 Mt CO_2_-eq year^−1^. The new supply mix further avoids 19 Mt CO_2_-eq year^−1^ over the whole life cycle by partially replacing the Russian gas with LNG and piped natural gas from the southern corridor. This replacement results in less pronounced upstream methane leakages, which offset the additional energy requirements of LNG in terms of GHG emissions (Figures S5 and S6).

We find that the first year of the crisis (Aftermath scenario) led to 0.92 Gt CO_2_-eq year^−1^, resulting in an 8.6% reduction in GHG emissions compared with the Base scenario (Figure 2B). Here, the increased natural gas demand in the power sector due to reduced nuclear and hydropower availability contributed to an additional 60 and 32 Mt CO_2_-eq year^−1^, respectively. Augmented coal-fired power plant hours and the increased use of oil for industrial heating resulted in an additional 16 and 6 Mt CO_2_-eq year^−1^, respectively.

Similar to the REPowerEU scenario, the new supply mix in the Aftermath scenario reduced impacts by 21 Mt CO_2_-eq year^−1^. The record wind and solar deployment level that decreased natural gas demand in electricity production by 11 bcm was responsible for reducing emissions by 14 and 12 Mt CO_2_-eq year^−1^, respectively. Increased efficiency in industry and households as well as the deployment of heat pumps led to emissions reduction of 7, 9, and 2 Mt CO_2_-eq year^−1^. On the other side, the main contributors to emissions reduction included electricity savings (41 Mt CO_2_-eq year^−1^), industrial production curtailment (36 Mt CO_2_-eq year^−1^), reduced individual heating due to mild winter (44 Mt CO_2_-eq year^−1^) and behavioral change resulting from public campaigns and high gas prices^40^ (14 Mt CO_2_-eq year^−1^).

Monte Carlo simulations considering uncertain life cycle inventory (LCI) data modeled using normal and lognormal distributions^41^ reveal that REPowerEU and Base scenarios are quite similar in the face of uncertainties. More specifically, we obtain near-zero *p*-values (Figures S7–S9) implying a statistically significant mean distinction between these scenarios. However, the probability of GHG emissions reduction from the former scenario relative to those in the latter is ca. 73% (Figure 2C), deemed inconclusive as it is closer to a coin toss than to 100%. On the other hand, we show using the same streamlined uncertainty analysis that the 86 Mt CO_2_-eq year^−1^ cut achieved in the Aftermath scenario is robust with all the 1000 Monte Carlo simulations leading to consistent impact reduction compared to the Base scenario (Figure 2D).

#### Carbon budgets would still be substantially transgressed by the natural gas consumption for energy systems

The life cycle CO_2_ emissions associated with natural gas supply and consumption in the Base scenario (0.89 Gt CO_2_, Table S4) largely surpass the EU’s carbon budgets consistent with a high likelihood of limiting global warming to 1.5°C (399% of the budget) and 2.0°C (139% of the budget), as in Figure 2E. The short-term policy actions in the REPowerEU plan (0.90 Gt CO_2_, Table S5) and the Aftermath (0.83 Gt CO_2_, Table S6) scenarios would still account for 403 and 373% of the 1.5°C budget and 140 and 130% of the 2.0°C budget, respectively. These transgression levels are computed by comparing the life cycle CO_2_ emissions of the EU natural gas use for energetic purposes to the CO_2_ emission budget as published in the Sixth Assessment Report (AR6) of the Intergovernmental Panel on Climate Change (IPCC).^42^ Since these are cumulative budgets allowed until the end of the century, they are here annualized and downscaled following the egalitarian principle^43^ to be compatible with our yearly and EU-wide system (see Method details section).

Consistent transgression results are observed if a fixed yearly carbon budget is employed and allocated by the population of the respective year, ranging from 295 to 319% of the 1.5°C budget and 103 and 111% of the 2.0°C budget (Figure S10). Notice that this approach results in a higher per-capita carbon budget for individuals today compared to future years, as the yearly carbon budget remains constant while the population increases over time.

Monte Carlo simulations show that LCI uncertainties would not alter substantially the transgression levels, with quartile coefficient of dispersion values between 3.6 and 4.6% (Tables S4–S8). Accordingly, besides all anthropogenic sources of GHG emissions, the natural gas sector alone transgresses the EU’s allocated annual emissions, shedding light on the importance of drastic changes as soon as possible. By comparing on a yearly basis, we show how distant the EU’s natural gas sector and the immediate action to manage the crisis to enhance energy security are from reaching emission levels that can guarantee agreed climate targets.

### Natural gas crisis management could lead to significant burden shifting

To shed light on the broader environmental implications of the three scenarios, we next study 15 additional impact categories based on the Environmental Footprint (EF) method v3.1 (explained in detail in Methods S2 of the SI). We employ a streamlined uncertainty analysis to compare the potential increase or reduction of impacts in the REPowerEU and Aftermath scenarios compared to the Base scenario. Specifically, we consider that those categories with probabilities of increased impacts between 25% and 75%, or *p*-values above 0.05, lack enough statistical evidence to conclude that one scenario is worse than the other under uncertainty in LCI data^44^ (see STAR Methods section for a detailed explanation).

**Figure 3:**
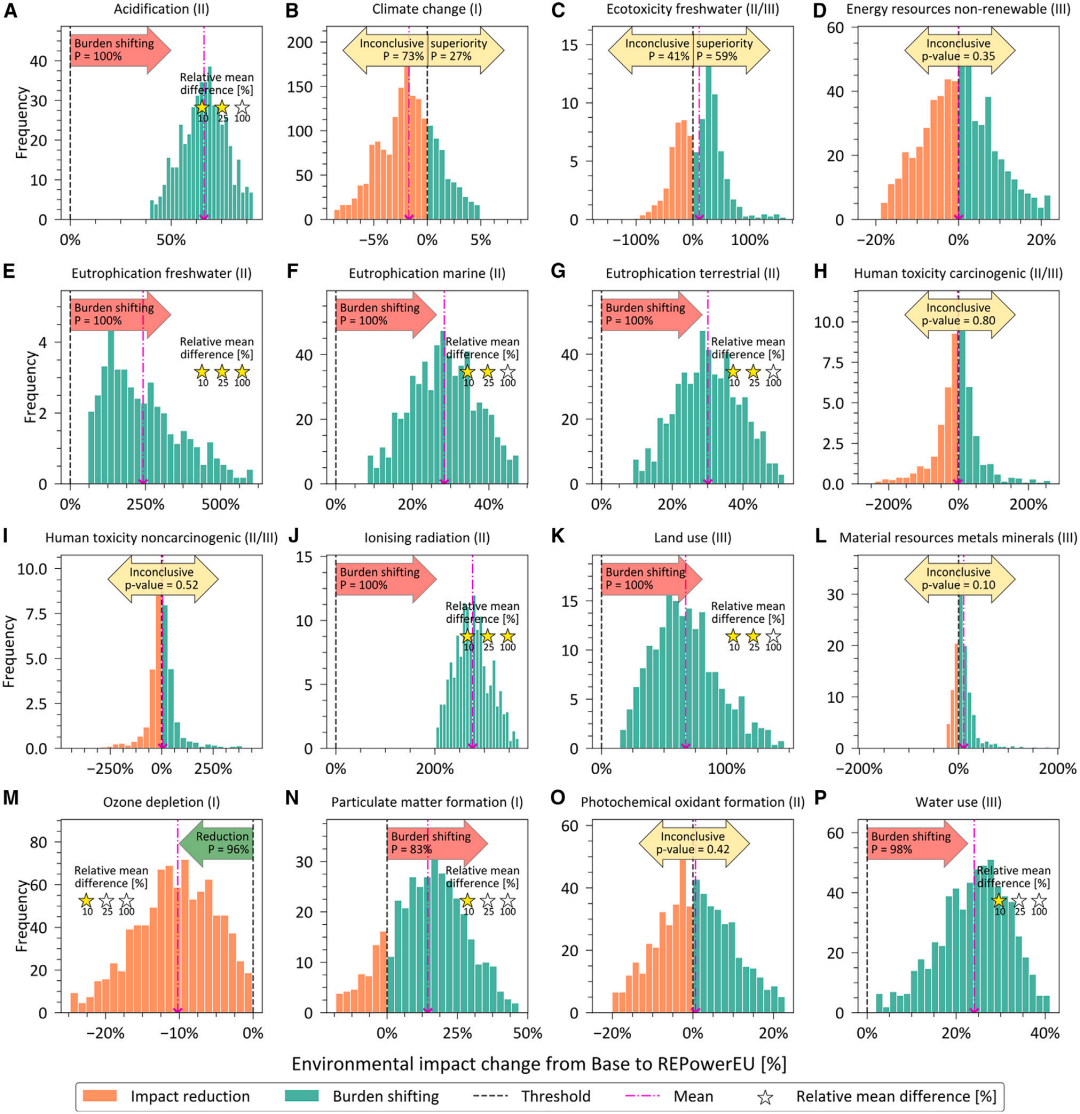
Statistically significant life cycle environmental impact difference between Base and REPowerEU scenarios (A–P) histograms depict the spread of environmental impact disparities between the Base and REPowerEU scenarios (impact of the latter minus the impact of the former across samples), considering the stochastic outcome of 1000 Monte Carlo simulations (Methods S1 in the SI). The selected 16 environmental impact categories are based on climate change (100-year GWPs as published in the AR6 of the IPCC^42^) and the additional 15 categories from the EF method v3.1 recommended by the European Commission.^45^ Here, positive values indicate potential burden shifting, while negative values imply impact reduction. The close-to-zero *p*-value rejects the null hypothesis of the paired samples having identical means (i.e., the scenarios are distinguishable), while high values indicate that there is little statistical difference between the scenarios (i.e., they are statistically indistinguishable). The probabilities (P in the figure) represent the likelihood of impact reduction (to the left) or burden shifting (to the right) in the alternative scenario (REPowerEU) compared to the Base case. One, two, and three colored stars represent the relative mean difference in absolute value from Base to REPowerEU to be at least 10, 25, and 100%, respectively, indicating, qualitatively, the amount of impact increase or decrease across categories. See also Figures S7–S9.

We find that the short-term measures in the REPowerEU plan would cause some statistically significant collateral damage via burden shifting (GHG emissions slightly decline at the expense of worsening other impacts) in eight categories, with impact increases above 100% in freshwater eutrophication and ionizing radiation and between 10 and 100% in acidification, marine and terrestrial eutrophication, land use, particulate matter formation, and water use (Figure 3). The only impact category benefiting in the REPowerEU scenario with statistical significance would be ozone depletion (12% reduction over the Base). The change in impacts from Base to REPowerEU in climate change, freshwater ecotoxicity, non-renewable energy resources use, carcinogenic and noncarcinogenic toxicity, metal and mineral resource consumption, and photochemical oxidant formation are regarded as inconclusive (Figures S7–S9).

**Figure 4:**
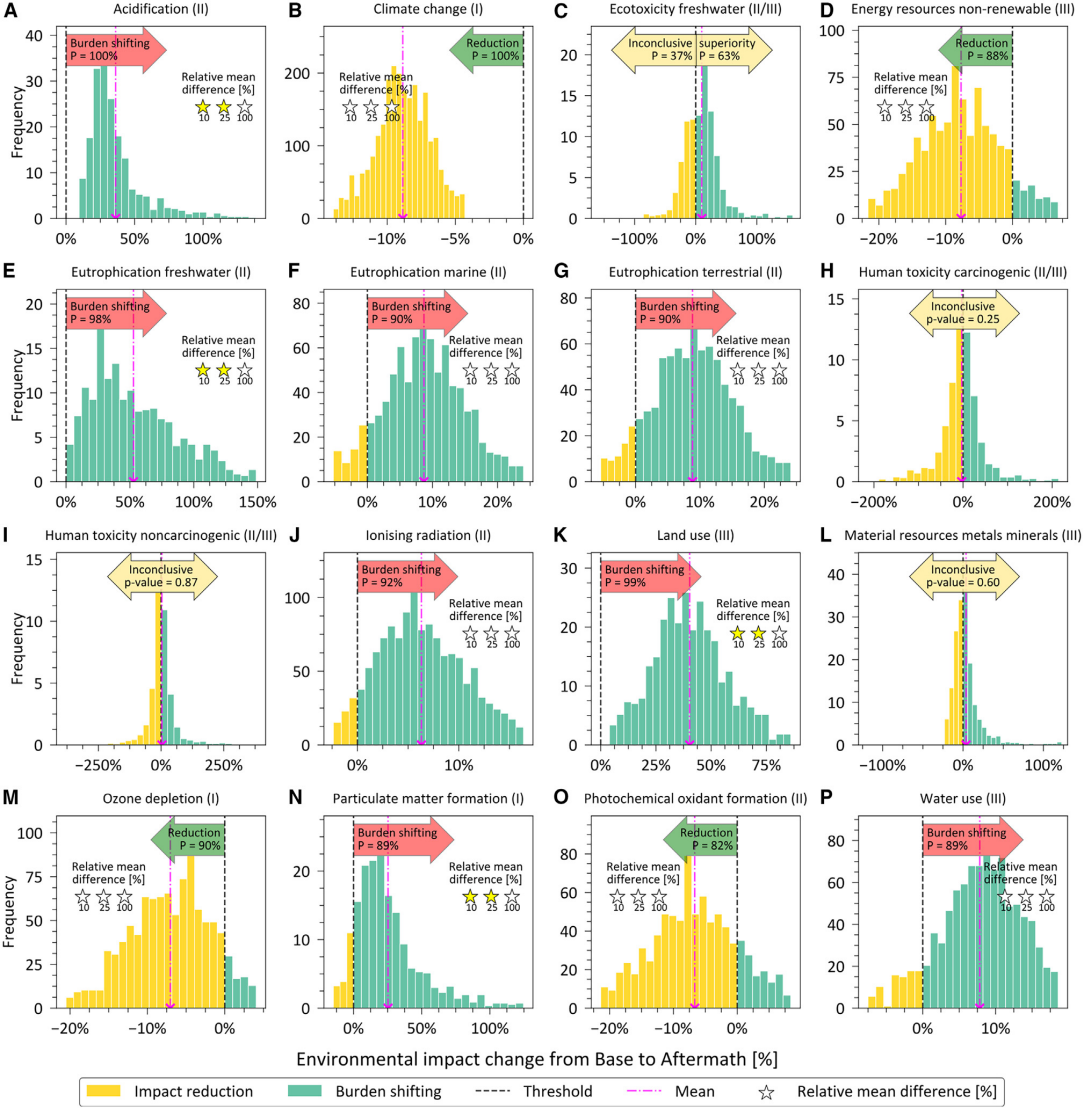
Statistically significant life cycle environmental impact difference between Base and Aftermath scenarios (A–P) histograms depict the spread of environmental impact disparities between the Base and Aftermath scenarios based on the 16 impact categories including climate change (100-year GWPs as published in the AR6 of the IPCC^42^) and the additional 15 categories from the EF method v3.1 recommended by the European Commission^45^ and considering the stochastic outcome of 1000 Monte Carlo simulations (Methods S1 in the SI). See the caption of Figure 3 for the interpretation of this figure. See also Figures S7–S9.

For the Aftermath scenario, we identified burden shifting to the same categories as in the REPowerEU scenario (Figure 4). However, here the intensity of the impact increase is at most 66% in freshwater eutrophication. On the other hand, besides a statistically significant reduction in GHG emissions as explained before, we also find a decrease in non-renewable energy resources use, ozone depletion, and photochemical oxidant formation of at most 9%.

We note that uncertainties in LCAs mostly arise from the LCI data and the impact assessment models. Because probabilistic information on the characterization factors used to translate emissions into potential impacts is missing, we studied the robustness of our results using Monte Carlo on uncertain LCI data (Methods S1 in the SI). Moreover, we provide in turn a qualitative discussion of the implications of uncertainties in the characterization factors. For the latter, we follow the recommendation level (or quality level) provided by the European Commission^46^^,^^47^^,^^48^ (Table S19). In essence, different levels of reliability were defined for each impact category based on the robustness of their impact assessment model. We observe that, except for land and water use, burden shifting occurs in more reliable categories (I and II quality levels).

#### Coal is the main root cause of environmental collateral damage

**Figure 5:**
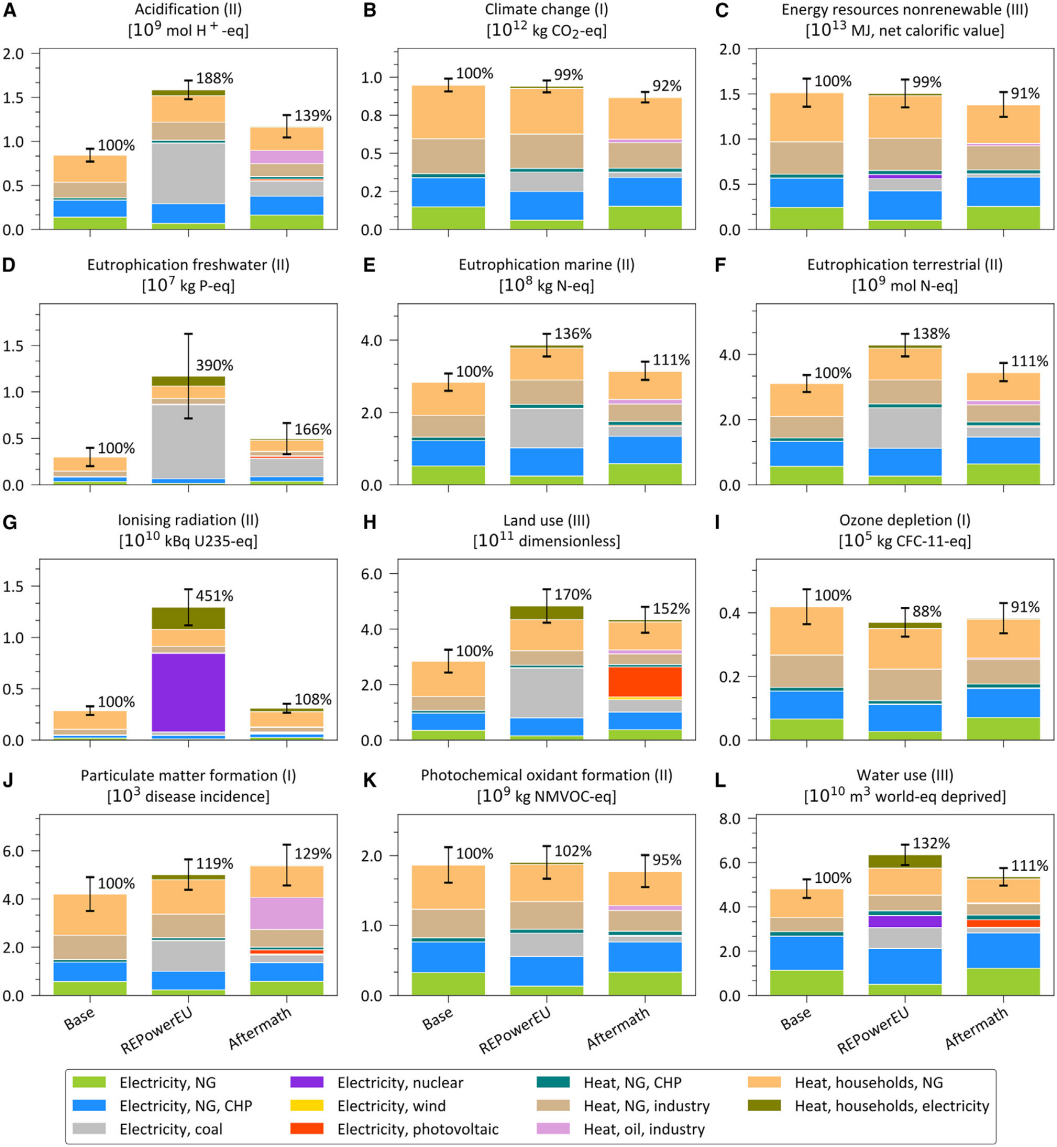
Breakdown of life cycle environmental impacts of restructuring the EU’s natural gas supply and consumption (A–L) show the contribution to the 12 impact categories out of climate change (100-year GWPs as published in the AR6 of the IPCC^42^) and the additional categories included in the EF method v3.1 recommended by the European Commission,^45^ in which there is statistically significant burden shifting or impact reduction. An increased use of coal-fired power plants is the major driver of this collateral damage. CHP: combined heat and power. NG: natural gas. Error bars indicate the quartile coefficient of dispersion (Methods S1). See also Figure S4 and Tables S4–S8.

A temporary turn back to coal-fired power plants would be the major driver of these unwanted side effects, as shown in the breakdown of impacts by energy type (electricity or heat) and energy source (natural gas, coal, renewables, etc.) for the three scenarios in Figure 5. More specifically, the life cycle impacts of coal-based electricity production are the main reason for increased acidification, eutrophication (freshwater, marine, and terrestrial), land use, particulate matter formation, and water use.

The impact increases in acidification, marine and terrestrial eutrophication, and particulate matter formation would be due to direct emissions from coal combustion in electricity production (e.g., SO_2_, NO_x_, fine particulate matter, and NH_3_). The treatment of spoil from coal mining would result in long-term phosphate emission to groundwater potentializing freshwater eutrophication. Also, coal mining would be responsible for increasing land use (mainly due to the land occupation from mining dumps and mineral extraction sites), water use, and particulate matter formation (from fine particulate matter, SO_2_, and NO_x_ emissions due to electricity production and blasting). Delaying the phase-out of nuclear power plants would exacerbate the ionizing radiation potential, which was rather low in the EU natural gas sector prior to the crisis, due to radioactive emissions from Radon-222 and Carbon-14 in the treatment of tailing from uranium milling as well as spent nuclear fuel. The life cycle impacts of the broader utilization of heat pumps for heating households and the service sector (e.g., in ionizing radiation and land and water use) would depend tightly on the electricity mix used to power them.

In the Aftermath scenario, oil combustion for industrial heating also contributed to exacerbated acidification, terrestrial eutrophication, and particulate matter formation due to emissions of SO_2_, NO_x_, and fine particulate matter to the air. The accelerated deployment of renewables in the first year of the natural gas crisis management, specifically from photovoltaic solar power, would have some negative effects on both land use due to mounting system production and water use from silicon production.

### Pinpointing the most critical impacts of EU’s natural gas crisis management

To better interpret the severity of burden shifting, we compare the impacts of each scenario with recently established annualized thresholds of the Earth’s carrying capacity^49^ based on the Planetary Boundaries framework^50^^,^^51^ (Table S18). In essence, burden shifting toward impact categories closer to their maximum allowable limits, which are established from the Earth’s ecological budget and following some downscaling principles, should be considered more critical.

**Figure 6:**
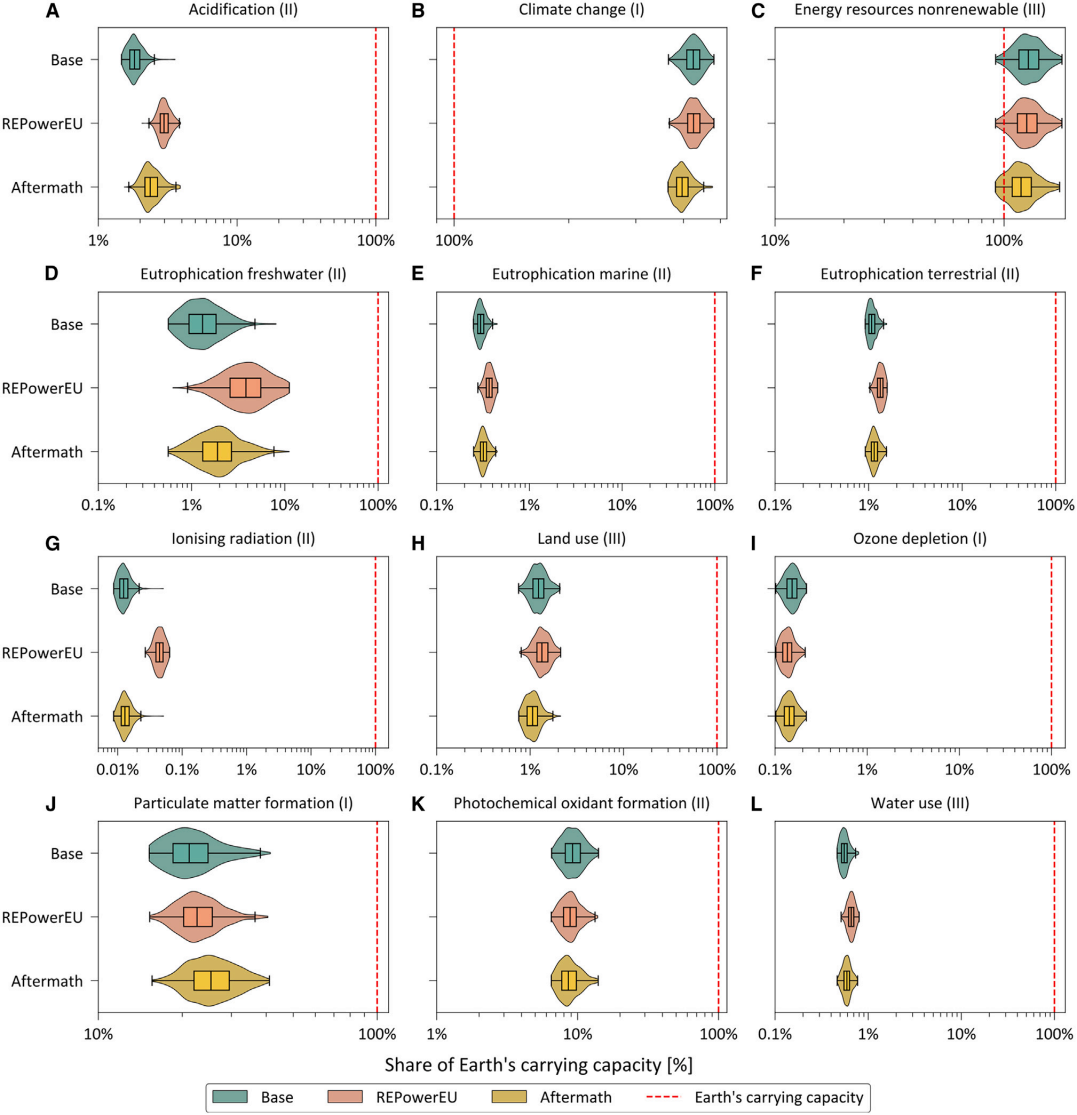
Life cycle environmental impacts of restructuring the EU’s natural gas supply and consumption compared against the Earth's ecological limits (A–L) show the contribution of the natural gas sector across scenarios to the EU’s ecological limit derived from the yearly global budget^49^ downscaled to the EU’s population following the egalitarian principle in 12 impact categories, where there is statistically significant burden shifting or impact reduction. The most relevant categories from an ecological budget transgression perspective correspond to climate change (373–403% of the maximum allowable impact), non-renewable energy resource consumption (101–111%), particulate matter formation (13–17%), and photochemical oxidant formation (ca. 7%). Notice here that climate change and land use impacts are calculated with life cycle CO_2_ emission and soil erosion methods, respectively, for a fair comparison with ecological budgets (see Table S18 for more detail). See also Figures S11–S13.

Assigning shares of the annualized global ecological capacity based on the EU’s population (egalitarian principle)^43^ to all anthropogenic emissions, we find that the most relevant collateral damage in the REPowerEU scenario (i.e., the whole natural gas sector including the short-term measures proposed in the REPowerEU plan) corresponds to particulate matter formation (16% of the maximum allowable impact), freshwater eutrophication (3%), and acidification (2%) (Figure 6; Figures S11–S13). Similarly, for the Aftermath scenario, the most relevant collateral damage occurs in particulate matter formation (17%) and acidification (2%). The first year of the crisis management led to a statistically significant impact reduction in some categories, with transgression levels of 101% in non-renewable energy resources use, 7% in photochemical oxidant formation, and close to 0% in ozone depletion. The impacts of the natural gas sector remain high on non-renewable energy resources consumption (from 101 to 111%), freshwater ecotoxicity (from 10 to 15%), material resource consumption (from 5 to 6%), and photochemical oxidant formation (around 7%), as in Figure S11, despite not involving burden shifting from Base to REPowerEU or Aftermath for these categories. On the other hand, the significant burden shifting to ionizing radiation, especially from considering additional nuclear power in the REPowerEU scenario (351% increase from Base), would have a negligible contribution to the EU’s ecological limit for this category because of initially low impacts.

We note that the identified statistically significant burden shifting, regardless of the share of the budget occupied, might be of concern for various reasons. First, the egalitarian downscaling provides a threshold for all anthropogenic emissions. Therefore, the low impacts of the natural gas sector do not imply that the global threshold will be met. Moreover, the robustness of assessing life cycle environmental impacts against the Earth's carrying capacity can be affected by temporal constraints of LCA, e.g., the lack of time dimension in the LCI models,^52^ and the absence of spatial granularity in some impact categories, e.g., land and water use, and eutrophication in different mediums.^53^ Nevertheless, we propose here the use of ecological limits to prioritize efforts and concerns in terms of environmental impact categories beyond climate change.

### Most effective measures to reduce the EU’s natural gas consumption

**Figure 7:**
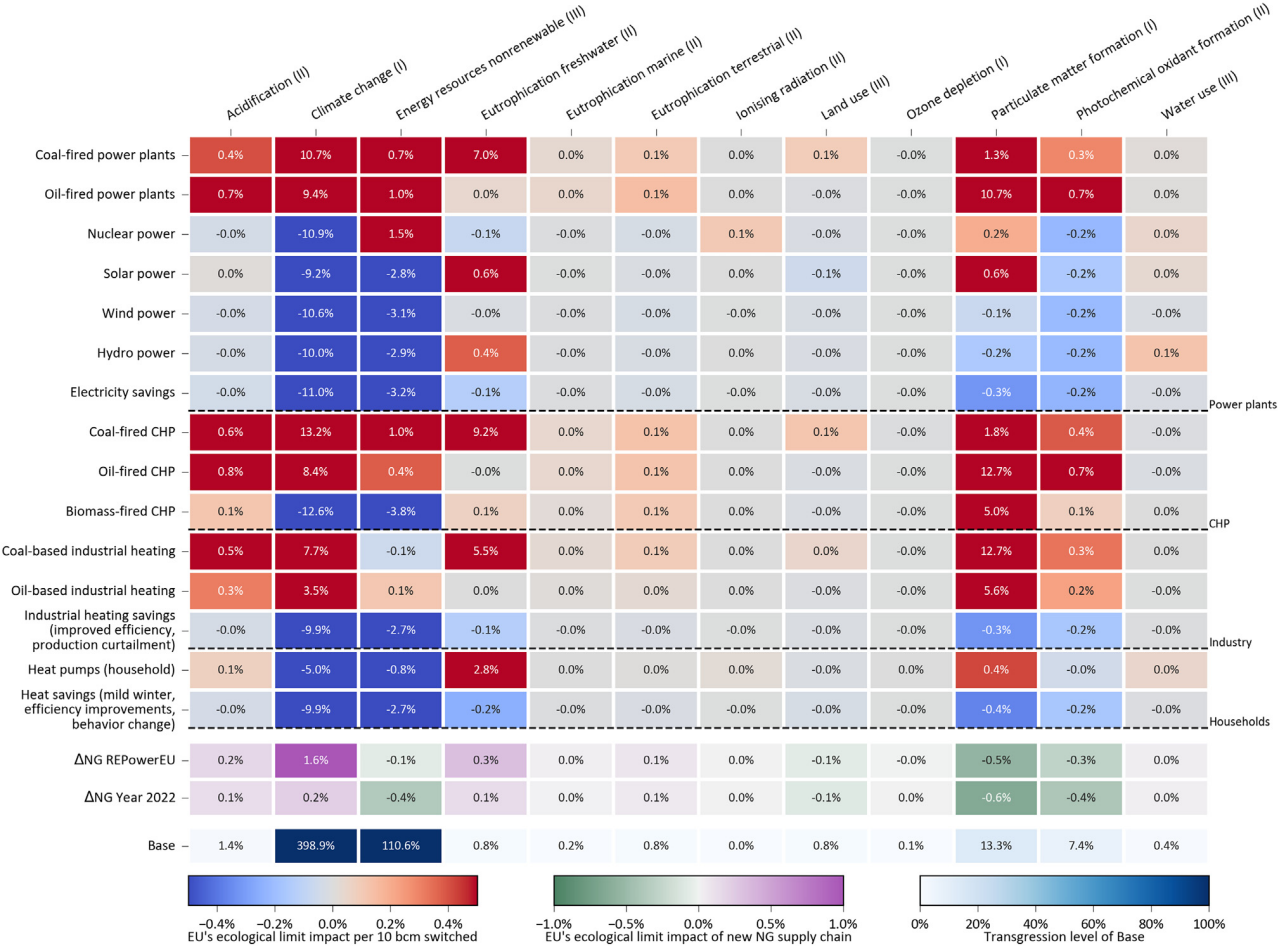
Life cycle environmental impacts on the EU’s share of the Earth’s ecological limits of individual measures to reduce natural gas consumption The top color map shows the transgression level impacts caused by switching 10 bcm of natural gas to an equivalent amount of other energy sources on a yearly basis. For example, switching 10 bcm of natural gas from the power plants to an equivalent amount of electricity production in coal-fired power plants would increase the transgression of the whole yearly Earth’s carrying capacity downscaled to the EU population by 10.0%. Notice that the color map is cut off at −0.5 and 0.5% to show trends in categories with impact values that are orders of magnitude lower than climate change. The second color map displays the same information but for the whole supply side measures (i.e., gas suppliers’ diversification) for both REPowerEU and Aftermath scenarios. The bottom color map provides the yearly transgression level from the natural gas sector before the crisis of 2022. CHP: combined heat and power. See also Figure S14.

Our previous results indicate that the EU’s efforts to reduce Russian gas imports had a negligible impact on GHG emissions while potentially resulting in burden shifting. To pave the way for a more sustainable restructuring of the EU’s energy system, here we investigate the most effective measures to reduce natural gas consumption. Figure 7 displays the impacts of replacing 10 bcm of natural gas with an equivalent amount of other energy sources on the Earth’s ecological limits downscaled to the EU (see STAR Methods section). Switching from natural gas to coal or oil in power generation and heat production in the industry emerge as the worst options across many impact categories. These measures to reduce natural gas consumption would lead to impacts representing 3.3–12.3% of the EU’s yearly carbon budget per 10 bcm replaced. In addition, such measures would cause a notable increase in other categories, such as acidification (up to 0.8% for oil-fired CHP), freshwater eutrophication (up to 1.1% for coal-fired CHP), and particulate matter formation (up to 12.4% for coal-based industrial heating).

Concerning the remaining measures, only energy savings could simultaneously reduce impacts in all categories, whereas the other measures would entail some environmental trade-offs. Shifting to nuclear, solar PV, wind, or biomass in power generation could reduce the impacts on the carbon budget by 9.2–10.7% per 10 bcm, albeit at the expense of worsening other impacts. Nuclear power would lead to burden shifting to non-renewable energy resource use (1.5% of the allowable impact limit per 10 bcm), biomass and solar PV to land use (6.4 and 0.5% per 10 bcm, respectively), solar and wind to mineral and metal resources use (1.8 and 0.2% per 10 bcm, respectively), and hydropower to water use (0.1% per 10 bcm). Wind and hydropower are the only options that would involve only minor trade-offs (below 0.2% of maximum allowable impacts per 10 bcm). Individual heating in households and the service sector can benefit considerably from heat pumps with a 4.2% reduction of the carbon budget transgression per 10 bcm switched, but leading to environmental trade-offs that are highly dependent on the electricity mix as discussed before. Considering the current electricity mix of EU countries, we find more relevant burden shifting to mineral and metal resources use (1.2% per 10 bcm), particulate matter formation (0.4% per 10 bcm), and freshwater eutrophication (0.3% per 10 bcm).

Analogously, we determined the relative change in environmental impacts due to the new EU natural gas supply mix, which involves mainly replacing imports from Russia with LNG from the United States. Supplier diversification could reduce impacts in categories benefiting from reduced methane leakage, i.e., climate change, ozone depletion, and particulate matter formation. In contrast, the main impact categories that would worsen due to the increase in LNG imports are acidification (0.1–0.2% of the maximum allowable limit in the REPowerEU and Aftermath scenarios, respectively), freshwater toxicity (2.3–3.1%), metal and mineral resources depletion (0.4–0.6%).

We have also assessed the relative change in impacts of replacing one bcm of natural gas with an alternative energy source to account for burden shifting and impact reduction in categories that do not extensively contribute to transgressing ecological limits (Figure S14). We show that replacing 24 bcm of natural gas in power plants with the corresponding energy-equivalent wind power production (instead of coal, as originally proposed in the REPowerEU) would reduce the burden shifting in particulate matter formation from 19% increase in comparison to Base (15% of the Earth’s carrying capacity downscaled to the EU population) to ultimately yield a net impact reduction of ca. − 4% (Figure S14). This change to the EU’s plan would also yield impact reductions in comparison with the Base case in photochemical oxidant formation (− 16%), climate change (− 14%), non-renewable energy resource consumption (− 9%), and terrestrial eutrophication (− 1%). However, it would still maintain some remaining collateral damage from the REPowerEU, i.e., acidification (from 88 to 8% increase from Base), freshwater eutrophication (from 290% to 35%), land use (from 70 to 20%), and water use (from 32 to 14%).

Finally, we go beyond assessing one policy recommendation to define optimal portfolios of measures to replace natural gas with current alternatives using an optimization model. LCA has been integrated into energy systems optimization models to study energy transition pathways,^54^^,^^55^ assessing the impacts of dynamic changes to the LCIs as well.^56^ Here, we present optimized portfolios of strategies to reduce natural gas consumption in the EU to cope with security of supply, building on the findings in Figure 7. In other words, we explore the potential environmental benefits that the energy crisis could indirectly bring by catalyzing the deployment of clean energy technologies. For that, we minimize the mean positive transgression level constrained to a natural gas demand reduction target of 50 bcm (see STAR Methods section for more information), finding that it can go from 38% (Base) to 30% (optimized scenario) by deploying wind power to replace this amount of natural gas (Table S9). For a more ambitious natural gas reduction target of 150 bcm, wind power (247.4 TWh), biomass-fired CHP (309.6 TWh of electricity), and heat pumps (267.2 TWh) would reduce the carbon budget transgression to 243%, while reducing the mean positive transgression level to 25% (Table S9). The remaining high transgression levels would be in non-renewable energy resources use (66%), particulate matter formation (49%), freshwater ecotoxicity (13%), freshwater eutrophication (10%), and mineral and metal resource use (9%). These results highlight that replacing natural gas with more sustainable energy resources can help reduce the pressure on different sustainability categories measured by mean transgression level. However, it would still fail to comply with the EU carbon emission budget.

## Discussion

The Russia-Ukraine war has led to a deep humanitarian crisis, high inflation, and energy and food security risk.^57^ The war has also caused structural changes in global supply chains. Here, we have shown that the short-term measures proposed in the REPowerEU plan to restructure the EU’s natural gas supply and consumption and reduce dependence on Russian gas would have a minor impact on GHG emissions, despite the relevant amount of coal considered in electricity production. However, these short-term policy measures, particularly the delayed phase-out of coal-fired power plants, may exacerbate other impacts beyond climate change, including acidification, eutrophication (freshwater, marine, and terrestrial), ionizing radiation, land use, particulate matter formation, and water use. Energy savings is the only measure that could simultaneously reduce all impacts; however, it would involve substantial changes in lifestyles and industrial activities to reduce natural gas demand with potential impacts on other sustainability dimensions (i.e., economic and social). Notably, the EU already transgresses the allocated share of the Earth’s carrying capacity for particulate matter formation and land use, while freshwater eutrophication is within the zone of uncertainty.^49^ Furthermore, high impacts in these categories may make it harder to attain various sustainable development goals (SDGs),^32^ i.e., SDG 3 on good health and well-being, SDG 6 on clean water and sanitation, SDG 14 on life below water, and SDG 15 on life on land.

The aftermath report from the IEA detailing the first year of the crisis following the geopolitical conflict reveals a significant 55 bcm reduction in natural gas demand within the EU,^9^ which represents the steepest drop in history, surpassing expectations outlined in the REPowerEU plan by 10%. The leading factors driving this downturn in natural gas demand and the success of managing the first year of the crisis include elevated gas prices, a mild winter, and prompt policy responses to the crises.^9^ Consequently, it resulted in natural gas supply diversification; fuel switching and reduced electricity consumption in the power sector; changes in behavior, fuel substitution, and issues of heating poverty among households; as well as production curtailment and shifts in fuel usage within the industrial sector.^9^ On the supply diversification side, from the 110 bcm reduction in Russian gas imports and the additional 50 bcm of LNG expected in the short-term of the REPowerEU, 98 bcm and 39 bcm were realized, respectively. On the other hand, the major discrepancies between the proposed policy actions and the first year’s aftermath include the smaller reliance on coal and nuclear power compared to the EU plan compensated by energy savings across sectors. These differences leveraged an 8.6% drop in GHG emissions for the first year of the crisis management and burden shifting to the same categories as in the short-term measures of the REPowerEU plan, although in a reduced magnitude.

Some of the drivers of reduced natural gas demand during the crisis might not be permanent (e.g., industrial production curtailments and electricity and heat savings). Hence, the full extent of the policy actions proposed in the REPowerEU plan for the short term may be essential in the following years should there be an imbalance in natural gas demand and supply. For instance, one of the most controversial measures in the plan is the temporary turn back to coal in electricity production. The plan specifies that up to 24 bcm of Russian gas could be replaced with coal,^8^ equivalent to generating an extra 117 TWh year^−1^ with existing coal-fired power plants. This represents a 28% increase in coal electricity production relative to 2021. Recent forecasts from the IEA confirm that coal electricity production increased in the EU in 2022.^58^ For example, 10 GW (GW) of coal-fired power plants re-entered the market in Germany.^58^ However, it has been followed by a record inland consumption decline in 2023 of ca. 22%^59^ as a consequence of the successful policy-making, especially the medium- and long-term measures of the REPowerEU kicking in and propelling the deployment of renewable energy.^60^ Nevertheless, we found that the GHG emissions from resorting to coal in the first year of the crisis have been offset with other measures (i.e., energy savings, deployment of wind and solar electricity capacity, and energy efficiency improvements in households and industry). Yet, increased reliance on coal is in conflict with the 1.5°C pathways assessed by the IPCC, which states that an 88% global reduction in coal electricity production would be needed from 2020 to 2030.^61^ Moreover, the collateral damage on other impact categories caused by coal mining and combustion should be a primary concern.

Increasing the imports of LNG played a pivotal role in the EU’s plans for securing natural gas supply. Our results show that partially replacing Russian gas with LNG would have a negligible impact on GHG emissions.^62^ Yet, this strategy can have broad consequences for the transition to a low-carbon economy because it may cause lock-in effects on natural gas infrastructure. The EU expanded its LNG import capacity by 40 bcm in 2023, with an additional 30 bcm expected to be operational in 2024.^63^ Germany is leading the investment in new LNG capacity, with forecasts indicating a 50 bcm expansion of regasification terminals by 2026.^64^ As the lifespan of regasification terminals can range from 20 to 30 years, the commission of new terminals can delay the deployment of renewable energies while hampering the achievement of climate targets,^26^ as the natural gas sector takes up alone around four times the EU’s yearly carbon budget, calling for a more aggressive step away from this energy resource toward cleaner alternatives. Notably, recent studies have shown that meeting the Paris Agreement requires that no new fossil infrastructure is commissioned while the existing infrastructure is retired early.^65^^,^^66^

Energy security could be enhanced by reducing Russian gas imports, yet this should not be done in a way that potentially hampers sustainable development. The full replacement of Russian gas with renewable energies would reduce GHG emissions, especially in Germany, Italy, and the Netherlands which imported, respectively, 55, 29, and 11 bcm in 2021.^67^ However, aligning the EU with the goal of limiting global warming well below 2°C would require a more aggressive stepping away from natural gas, as non-Russian suppliers accounted for the majority (ca. 62%) of the EU’s natural gas consumption.^1^ In the medium- and long-term, the REPowerEU aims for a large-scale deployment of wind and solar PV power, renewable hydrogen, heat pumps, energy efficiency initiatives, and biomethane.^8^ These measures appended to the short-term ones are expected to reduce natural gas consumption by 310 bcm by 2030,^8^ equivalent to ca. 81% of the natural gas consumed for energy purposes in the EU in 2021. These policy actions could substantially reduce GHG emissions by 2030, albeit they might not be exempt from burden shifting as demonstrated per technology in Figure 7. For instance, reducing natural gas demand for electricity production through the deployment of solar PV power could increase the impacts on freshwater eutrophication and particulate matter formation mainly from increased mining operations (Figure 7). Similarly, relying more on renewable hydrogen may cause unwanted human and ecotoxicity impacts that stem from the extraction and processing of metals for renewable energy infrastructure.^68^

Finally, this work provides policy-makers with comprehensive environmental impact estimations of measures to cope with the efforts of reducing natural gas consumption and consequent imports in the EU (Figure 7), which could serve as guidance to devise more effective long-term strategies. We show here the importance of considering the significant (statistically and relative to ecological limits) collateral damage that might be incurred when making systematic changes to energy systems. Additionally, we show that the natural gas crisis could catalyze the transition to clean energy technologies by fostering measures to reduce energy supply risk. The deployment of wind power to replace natural gas-fired power plants appears to be the most suitable strategy to curb GHG emissions while also reducing other impacts. Biomass-based CHP and heat pumps are also beneficial overall beyond GHG emissions; however, they are insufficient to keep these sectors within the EU’s ecological limits. Given the importance and magnitude of natural gas use in industrial heating (responsible for delivering 991 TWh of thermal energy), its replacement should be the focus of future efforts. All in all, keeping impacts across environmental sustainability categories within allowable limits in future endeavors to step away from natural gas would require optimized portfolios of technologies.

The main learnings and potential policy implications of this study can be summarized in three directions. First, we show the ability of the EU to develop short-term action plans to ensure energy security during energy crisis times without necessarily increasing GHG emissions. Therefore, to mitigate the controversy around temporarily reverting to high-emission energy sources (e.g., delaying the phase-out of coal), the potential for offsetting the increased impact by other measures (e.g., energy savings) needs to be rigorously analyzed and communicated. Second, these short-term plans may lead to substantial burden shifting, mainly driven by a temporal return to energy sources like coal. This aspect also deserves careful consideration and transparent communication. Finally, the methodological approach presented in this study, combining scenario analysis with LCA, could be extended and applied to assess the long-term strategies. We hope this discussion could open new avenues for future research to build on our modeling framework, addressing the broader impacts of further reducing the EU natural gas demand in the future.

### Limitations of the study

To provide more comprehensive and deployable pathways to reduce the EU’s reliance on natural gas while avoiding burden shifting, the current approach could be integrated into energy systems models. This would allow solving for optimized portfolios of measures exploiting regional advantages and taking into account limited resources (water, land, materials, etc.). However, this effort would require further research on regionalized characterization factors.^69^^,^^70^ Further research efforts are also needed on downscaling principles^43^ (e.g., considering regionalization and climate justice of carbon budget^71^^,^^72^) and spatially explicit limits on Earth-system processes.^53^ Another limitation of this work is the lack of a comprehensive list of sustainable alternatives to replace natural gas for energy applications in power generation, industry, and households. That would demand a thorough analysis of currently ready technologies and the ones under development and could be the focus of future research. Moreover, comparing optimized strategies to reduce natural gas demand further with current policies (e.g., medium- and long-term measures in the REPowerEU) and considering future projections could be the focus of subsequent studies. Notably, subsequent research efforts could assess the environmental impacts of changes in a future economy, for which prospective LCA would likely be required.^73^ It would also require careful analysis of modeling assumptions when it comes to emerging technologies. For instance, the deployment of renewable hydrogen is subject to uncertainties in terms of production technology and regionalization, storage, transportation, and final consumption.^74^ Additional limitations of the study are discussed in Methods S3 in the SI.

## Resource availability

### Lead contact

Further information and requests for resources should be directed to and will be fulfilled by the lead contact, Gonzalo Guillén-Gosálbez (gonzalo.guillen.gosalbez@chem.ethz.ch).

### Materials availability

This study did not generate new unique materials.

### Data and code availability

• This paper analyses existing, publicly available data, accessible in this paper supplemental information.
• All original code needed to reproduce the results presented in this study has been deposited at https://github.com/lf-santos/NG-2022crisis-LCA and is publicly available at https://doi.org/10.5281/zenodo.14163698.
• Any additional information required to reanalyze the data reported in this paper is available from the lead contact upon request.

## Acknowledgments

L.F.S. acknowledges the National Council for Scientific and Technological Development–CNPq (Brazil), process number 200305/2020-4.

## Author contributions

Conceptualization, L.F.S., R.I., and G.G.-G.; methodology, L.F.S., R.I., and G.G.-G.; investigation, L.F.S. and R.I.; writing – original draft, L.F.S., R.I., and G.G.-G.; writing – review and editing, L.F.S., R.I., N.M.C., and G.G.-G.; funding acquisition, L.F.S. and G.G.-G.; visualization, L.F.S.; supervision, G.G.-G.

## Declaration of interests

N.M.D. consults widely for a variety of public and private net zero transition stakeholders.

## STAR★Methods

### Key resources table

**Table:** 

REAGENT or RESOURCE	SOURCE	IDENTIFIER
**Deposited data**		

Ecoinvent life cycle assessment database v3.9.1 (cut-off system model)	Ecoinvent^41^	https://ecoinvent.org/
Eurostat complete energy balances	Eurostat^2^	https://doi.org/10.2908/NRG_BAL_C
Bruegel European natural gas imports dataset	Bruegel^1^	https://www.bruegel.org/dataset/european-natural-gas-imports

**Software and algorithms**		

Python v3.9.18	Python Software Foundation	https://www.python.org/
Brightway2 v2.4.3	Brightway LCA Software Framework^75^	https://github.com/brightway-lca/brightway2
Activity-Browser v2.9.0	LCA-ActivityBrowser^76^	https://github.com/LCA-ActivityBrowser/activity-browser
Wurst v0.3.3	POLCA^77^	https://github.com/polca/wurst
Presamples v0.2.8	PascalLesage^78^	https://github.com/PascalLesage/presamples
Pyomo v6.7.3	Pyomo^79^	https://www.pyomo.org/
Code to reproduce this study	This study	https://doi.org/10.5281/zenodo.14163698

### Method details

#### Scenario definition

We applied the standardised LCA methodology^80^^,^^81^ to quantify the potential environmental impacts of alternative scenarios for natural gas supply and consumption in the EU. The functional unit was defined as the EU’s annual consumption of natural gas-based electricity and heat in industry and households as they were before the invasion of Ukraine, i.e., 557 TWh of electricity, 213 TWh of heat from CHP, 991 TWh of heat in industry, and 1380 TWh of heat in households.^2^ The system boundaries included all the relevant impacts from the extraction, processing, distribution, and combustion of fossil fuels (e.g., natural gas and coal) as well as the manufacturing, operation, and final disposal of the required infrastructure (i.e., power plants, wind turbines, solar PV panels, boilers, etc.).

#### Life cycle inventory

The LCI database ecoinvent v3.9.1 (cut-off system model)^41^ was used to model the alternative scenarios (see Figure S15; Tables S10–S16 in the SI). Regarding electricity supply, ecoinvent’s inventories for stand-alone and CHP natural gas-fired, coal-fired, nuclear, wind, and solar PV power plants were used. More specifically, we created regional supply mixes for each electricity production option by considering the share of each EU Member State based on the electricity mixes available in the ecoinvent database. For example, natural gas combined cycle power plants in Italy contribute with ca. 5% of the electricity produced from natural gas in the EU. Heating was divided into heat from CHP, heat in industry, and heat in households. As for electricity production, we created regional heating mixes for the EU based on data available in the ecoinvent database. Finally, we also created a new inventory for the supply of high-pressure natural gas at the EU level. This activity contained the contribution share of each supplier, i.e., domestic production, imports by pipeline from Russia, Norway, Algeria, and Azerbaijan, and imports of LNG from the U.S., Qatar, Russia, Nigeria, and Algeria. Note that the share of each supplier was modified according to the scenario assessed.

An important aspect of our modeling approach is that we considered the interdependencies between the measures implemented in the scenarios as well as their impact on the other activities in the background system. This was achieved by relinking our tailored activities among them as well as to the other activities in the LCI database. This implies that if, for example, an activity requires natural gas from the EU market, this natural gas is supplied according to the supply mix defined in our scenario. Similarly, if an activity requires electricity from the EU mix, this mix has a higher or lower share of natural gas-fired, coal-fired or nuclear power plants depending on the scenario assessed. The systematic modification of the LCI database as well as the life cycle impact assessment (LCIA) calculations were performed with the open-source LCA software Brightway2^75^ and Wurst.^77^ The reader is referred to Methods S2 in the SI for more background information on LCIA calculation as well as the assumptions and interpretation of the impact assessment methods employed. Additionally, methodological assumptions and limitations of our analysis is discussed in Methods S3.

#### Climate change and carbon budgets

The climate change impacts assessment was carried out considering the 100-year GWPs published in the AR6 of the IPCC.^42^ We also calculated the life cycle CO_2_ emissions associated with natural gas supply and consumption against the IPCC AR6 carbon budgets consistent with a high probability (67%) of limiting global warming to 1.5°C and 2.0°C compared with pre-industrial level.^42^ Since the carbon budgets represent cumulative global CO_2_ emissions, they need to be downscaled to the EU level. Here, we employed the egalitarian principle, according to which the carbon budget is allocated on an equal share per capita basis.^82^^,^^83^ Similarly, to annualize the per capita carbon budgets consistent for the years between 2020 and 2100, we divided them by the cumulative population in the same period, achieving constant yearly per capita emission limits,^82^ such that

$$ICB\left[\frac{kg\;CO_{2}}{person\cdot year}\right]=\frac{{CB}_{2020-2100}}{\int_{2020}^{2100}Pop\left(t\right)\;dt}=\frac{{CB}_{2020-2100}\left[kg\;CO_{2}\right]}{{CPop}_{2020-2100}\left[person\cdot year\right]}$$

where ICB is the individual, yearly carbon budget, $CB_{2020-2100\;}$ is the global CO_2_ emission threshold to achieve some temperature increase goal with some likelihood, and $CPop_{2020-2100}$ is the cumulative world population between 2020 and 2100 (based on world population prospects from the United Nations^84^), see Table S17 in the SI. Notice that the yearly carbon budget per capita is the same for every person in the world from 2020 to 2100, which agrees with the egalitarian principle. In addition to climate change impacts, we assessed other 15 impact categories included in the EF method v3.1 recommended by the European Commission.^45^^,^^85^

#### Sensitivity analysis

We performed a sensitivity analysis to determine the relative change in environmental impacts caused by the supply and demand measures to cope with the shortage of natural gas imported from Russia. We calculated the difference in life cycle impacts that occurred by switching the equivalent electricity and heat production from 10 bcm of natural gas to the alternative technologies (15 options as presented in Figure 7) using the open-source Python package Presamples^78^ jointly with Brightway2^75^ (Table S20). The sensitivity analysis results, jointly with the environmental impacts of restructuring the natural gas supply mix (60 bcm from Russia replaced by LNG and piped gas from Azerbaijan), were divided by the EU’s ecological limits of each impact category (Table S18) to provide relative changes. It should be noted that because the model for calculating life cycle impacts is linear,^86^ the sensitivity information calculated for 10 bcm of natural gas can be extrapolated to larger amounts.

#### Optimisation analysis

We derived an optimisation model to determine optimal portfolios of policy recommendations based on different natural gas reduction targets. Departing from the sensitivity analysis results, we want optimise the mean positive transgression level (MPTL) across impact categories considering the natural gas demand reduction alternatives in Figure 7. As such, we define

$$MPTL=\frac{\sum_{l\in L}\;\max\left\{\sum_{r\in R}\left(d_{r}{\cdot \rho }_{r,l}\right)+\Delta_{l},0\right\}}{\left|L\right|}\text{,}$$

in which L is the set of 16 impact categories from EF v3.1, R is the set of 15 natural gas replacement alternatives (Figure 7), $d\in R^{15}$ are the decision variables accounting for the natural gas replacement to other technologies in bcm, $\rho \in R^{15\times 16}$ are the parameters with the sensitivity results of the impact of alternative r in EU’s ecological limit of category l per bcm, and $\Delta \in R^{16}$ are the parameters of the impacts of the new supply mix on the ecological limits. Specifically, MPTL is the sum of positive impacts (computed by applying the max operator between the impact and zero) of substituting natural gas by other energy sources across all impact categories divided by the number of impact categories. Reformulating MPTL with positive slack variables,^87^ $t\in R^{16}$, the resulting optimisation model is a linear programming problem as follows

(Equation 1) $$\begin{array}{cc} \min & \frac{\sum_{l\in L}e_{l}}{\left|L\right|}\\ s.t\text{.} & t_{l}=\sum_{r\in R}\left(d_{r}\cdot \rho_{r,l}\right)+\Delta_{l},\forall l\in L\\ & e_{l}\geq t_{l},\forall l\in L\\ & e_{l}\geq 0,\forall l\in L\\ & \sum_{r\in R}d_{r}=\bar{d}\\ & \sum_{r\in P}d_{r}\leq \bar{d_{P}},\sum_{r\in C}d_{r}\leq \bar{d_{C}},\sum_{r\in I}d_{r}\leq \bar{d_{I}},\sum_{r\in H}d_{r}\leq \bar{d_{H}} \end{array}$$

where $e\in R^{16}$ is the variable accounting for the absolute impact on the EU’s ecological limit in category l and $\bar{d}\in R$ is the natural gas demand reduction target. Additionally, d is constrained by the total amount of natural gas required by power plants (P⊂R), CHP (C⊂R), industry (I⊂R), and household (H⊂R), defined as $\bar{d_{P}}$, $\bar{d_{C}}$, $\bar{d_{I}}$, and $\bar{d_{H}}$, respectively. The solution of the optimisation problem 1, $d^{*}\in R^{15}$, defines the amount of natural gas in bcm that is replaced by each of the other technologies as in Figure 7 to minimise MPTL. This linear programming problem is modeled in Pyomo^88^ and solved with the solver CPLEX 22.1.1^79^, guaranteeing that the solution is globally optimal. Therefore, the resulting MPTL is the lowest possible value that could be attained, given the considered natural gas demand reduction target ($\bar{d}$) and set of alternative technologies (R).

### Quantification and statistical analysis

We generated 1000 Monte Carlo samples for both technosphere and biosphere data for each scenario, followed by subsequent LCIA calculations and the computation of key statistical metrics. Subsequently, statistical indicators were computed for scenario comparisons, including the *p*-value to study the statistical mean difference across scenarios and the probability of increased emissions (discernibility) for scenario superiority.^44^

We streamlined the scenario comparison process by first evaluating the *p*-value from the number of degrees of freedom (df=n−1) and the paired sample t-test (t) of equal means given by

$$t=\frac{\bar{d}}{\sqrt{\frac{s_{d}^{2}}{n}}}\text{,}$$

where $\bar{d}$ and $s_{d}$ are the average and standard deviation of the uncertain outcome difference between two scenarios, and n is the number of experiments. If the *p*-value exceeds 0.05, both scenarios are deemed to have indistinguishable mean values, implying no statistically meaningful difference. Conversely, if it is below 0.05, we proceed to assess scenarios based on the probability of increased emissions from Base (B) to some alternative scenario (A) denoted P(A>B) using the discernibility analysis for dependent datasets,^89^ such that

$$P\left(A>B\right)=\frac{1}{n}\sum_{i=1}^{n}\Theta \left(a_{i}-b_{i}\right)\text{,}$$

where Θ is the Heaviside step function (returns 1 if its argument is positive and 0 otherwise), $a,b\in R^{n}$ are the Monte Carlo simulation outcomes for the alternative and Base scenarios, respectively (i.e., impact values of each alternative in simulation i). A probability exceeding 75% denotes a significant emission increase, while a value below 25% indicates a significant emission reduction. For probabilities between 25% and 75%, the statistical significance of one scenario being superior over the other is considered inconclusive. See Methods S1 in the SI for more details on the uncertainty analysis.

## Footnotes

Supplemental information can be found online at https://doi.org/10.1016/j.isci.2024.111575.
