# Supplementary material for: Environmental impacts of restructuring the EU’s natural gas supply and consumption: Learnings from the 2022 energy crisis
Source: iScience. 2024 Dec 12;28(1):111575. doi: 10.1016/j.isci.2024.111575 (PMC11763217; doi:10.1016/j.isci.2024.111575)
Supplement: Document S1. Figures S1–S15, Tables S1–S20, and Methods S1–S3 [file mmc1.pdf]

iScience, Volume *28*

Supplemental information

Environmental impacts of restructuring the EU's natural gas supply and consumption: Learnings from the 2022 energy crisis

Lucas F. Santos, Robert Istrate, Niall Mac Dowell, and Gonzalo Guillén-Gosálbez

Supplemental Figures

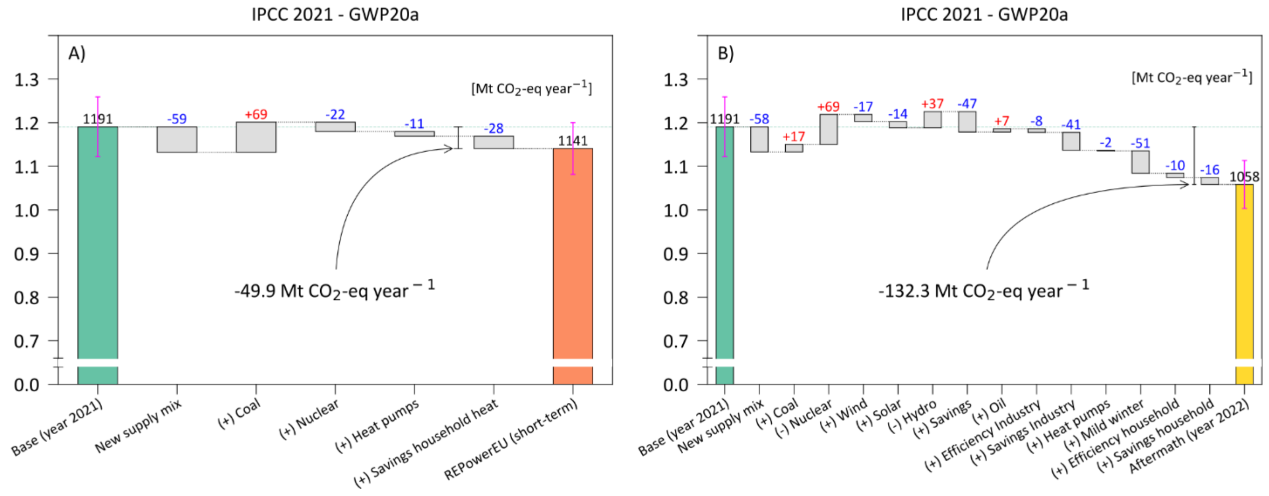

Figure S1. Life cycle climate change impacts of the REPowerEU (A) and Aftermath (B) scenarios, considering the 20-year global warming potentials GWPs, related to Figure 2. The 20-year GWPs account for the impacts in the short term, thus prioritising gases with shorter lifetimes, i.e., methane. We found that the Base scenario would result in 1.12 Gt CO_2-eq year^{-1}, while the REPowerEU plan would reduce greenhouse gas (GHG) emissions by 50 Mt CO_2-eq year^{-1}, equivalent to a 4.2% reduction over the Base. The Aftermath scenario entails a 132 Mt CO_2-eq year^{-1} reduction from the pre-invasion levels, totalising 1.06 Gt CO_2-eq year^{-1}. These results show a consistency with GWP over 100 years and, therefore robustness of GHG emission impacts.

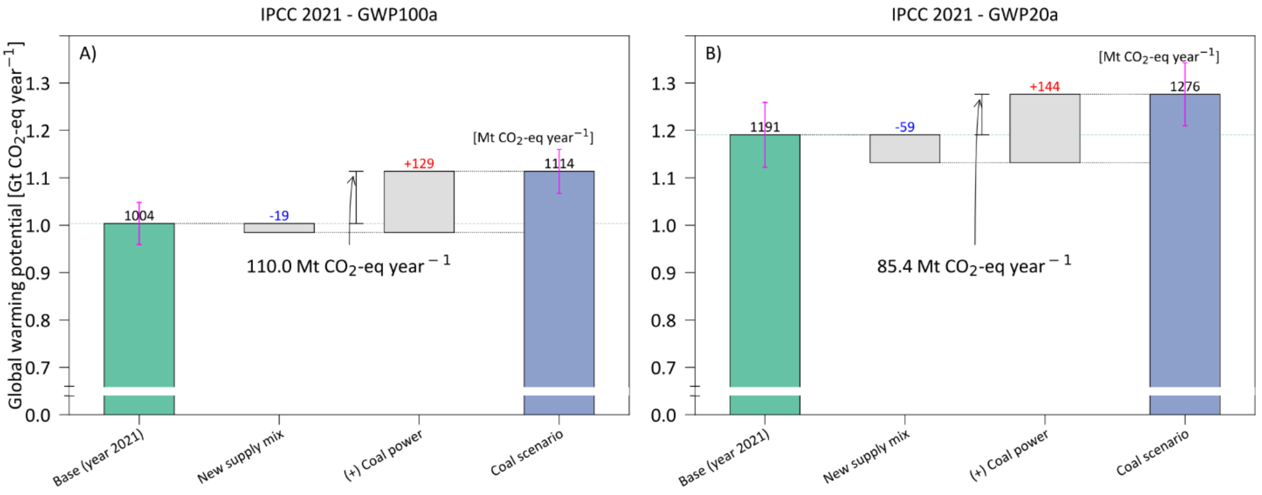

Figure S2. Life cycle climate change impacts of the Coal scenario, considering the (A) 100-year and (B) 20-year global warming potentials (GWPs), related to Figure 2. The Coal scenario considers compensating 50 bcm of natural gas using coal in the power sector. Such an extreme, hypothetical scenario provides further insights into the analysis and approximate an upper bound on the environmental impacts in the first year of the crisis. The alternative Coal scenario shows that switching to coal to a larger extent (the energy equivalent of 50 bcm of natural gas) would increase GHG emissions by 11% over the Base.

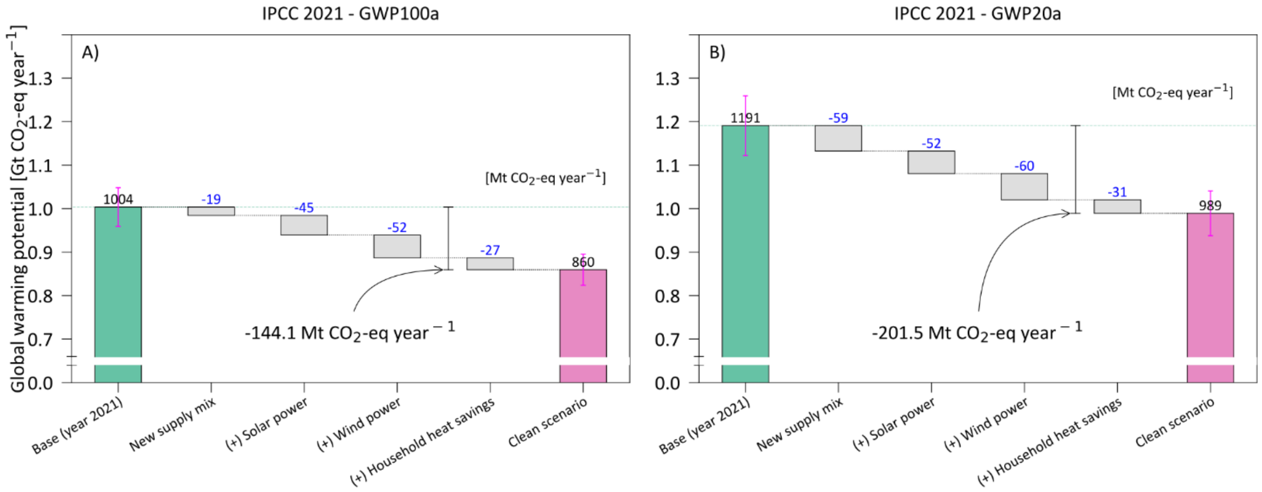

Figure S3. Life cycle climate change impacts of the Clean scenario, considering the (A) 100-year and (B) 20-year global warming potentials (GWPs), related to Figure 2. The Clean scenario reduces 10 bcm via heat savings in households while replacing 20 bcm in power generation with wind power and 20 bcm with solar photovoltaic (PV) power. The equal share of wind and solar power was defined in accordance with Pedersen et al. [S1], who found similar growth rates for both renewables during the decarbonisation of the European energy system assuming a reduced natural gas availability. Such a hypothetical scenario provides further insights into the analysis and approximate a lower bound on the environmental impacts in the first year of the crisis, which does not consider the deployment time of renewable. The new supply mix, energy savings, and a higher reliance on wind and solar PV power (Clean scenario) would reduce emissions by 14% over the Base, equivalent to 144 Mt CO_2-eq year^{-1}.

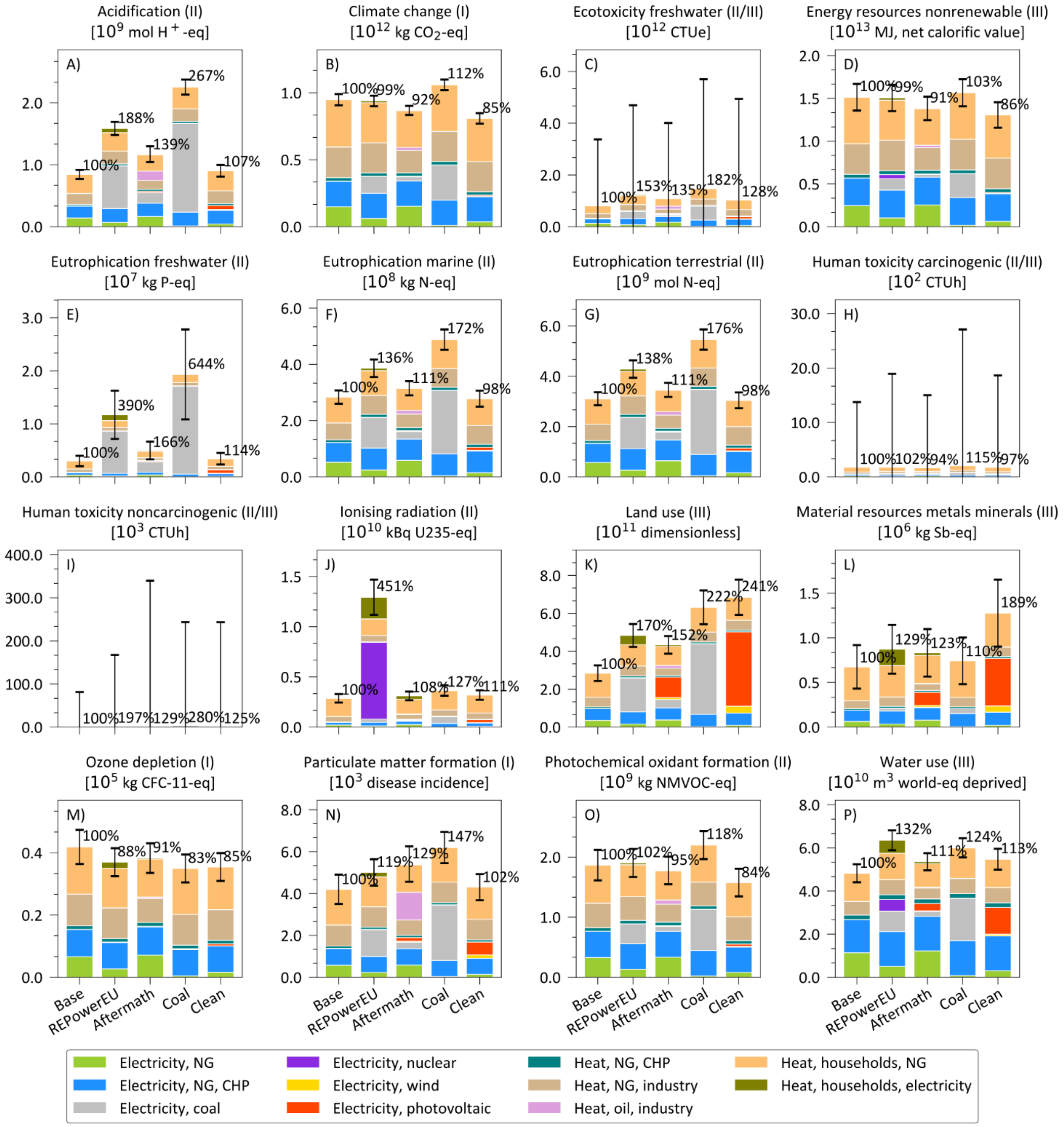

Figure S4. Life cycle environmental impacts breakdown of restructuring the EU's natural gas supply and consumption to reduce 110 bcm of Russian gas across scenarios, related to Figure 5. (A-P) show the contribution to 16 impact categories out of climate change (100-year GWPs as published in the AR6 of the IPCC [S2]) and the additional 15 categories included in the EF methods v3.1 recommended by the European Commission [S3]. The Coal scenario would substantially exacerbate impacts linked to coal combustion and mining, while the Clean scenario would also lead to burden-shifting in some categories. Notably, replacing 40 bcm of natural gas with wind and solar PV power could reduce impacts on climate change, non-renewable energy resources utilisation, ozone depletion, and photochemical oxidant formation. However, deploying additional PV power would worsen especially land use (from land occupation and transformation to industrial areas in PV mounting system production); metal and mineral resources consumption (from Tellurium, Gold, Silver, and Cooper use in PV panel and inverter production and PV electric installation); and water use (from silicon production for the PV cell). CHP: combined heat and power. NG: natural gas.

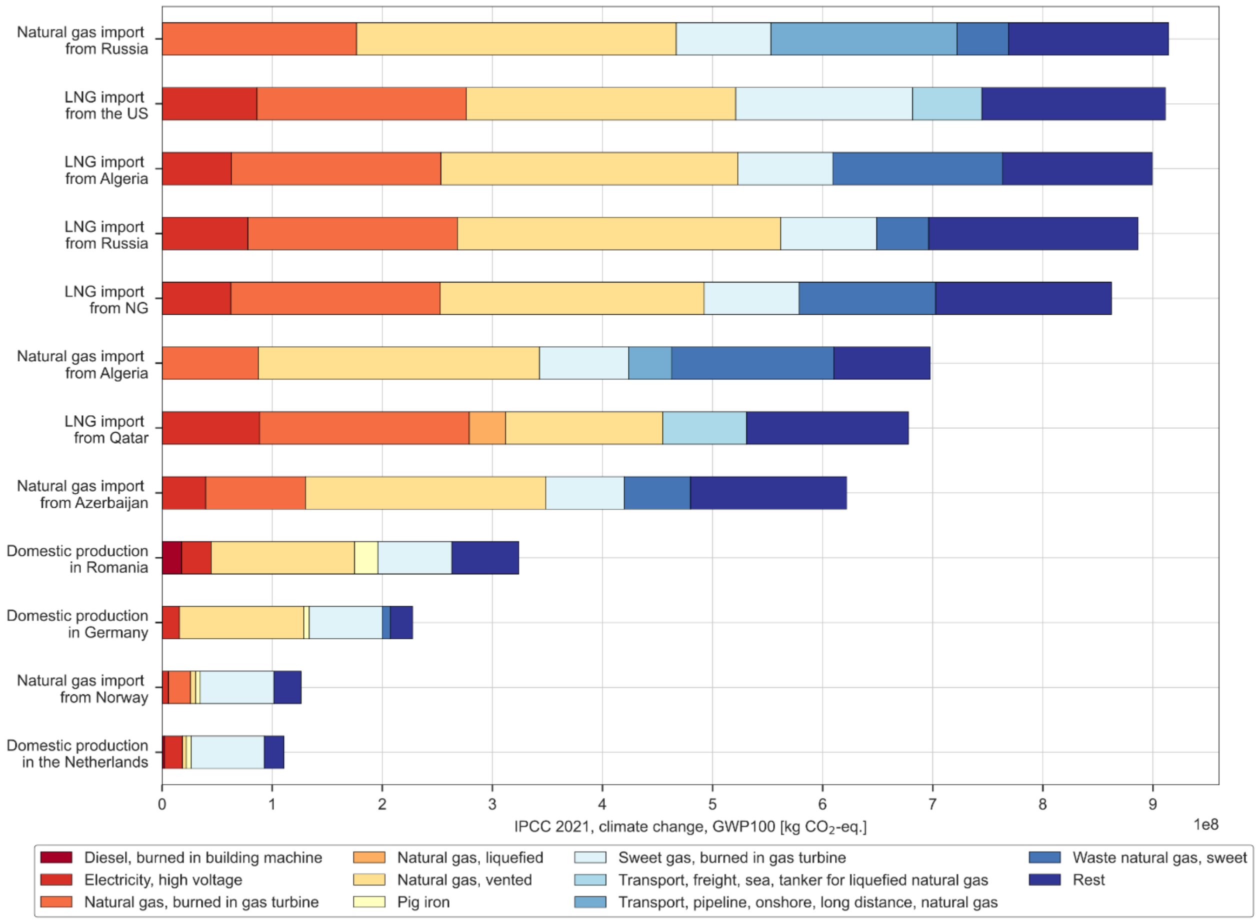

Figure S5. Life cycle carbon intensity of importing natural gas from the main suppliers of the EU in gaseous phase and as LNG broken down by process contribution, related to Figure 2. The functional unit considered is 1 bcm of natural gas imported in the gaseous phase in pipelines or as LNG followed by its regasification from the main suppliers to the EU. The LCIA method employed was the IPCC 2021, 100-year averaged GWP in kilogram of CO_2 equivalents (kg CO_2-eq) [S2]. These results show that switching natural gas imports from Russia to LNG from the US and piped gas from Azerbaijan is beneficial from a climate change point of view, especially for the reduced natural gas leakage and the reduced transport emission concerning the latter.

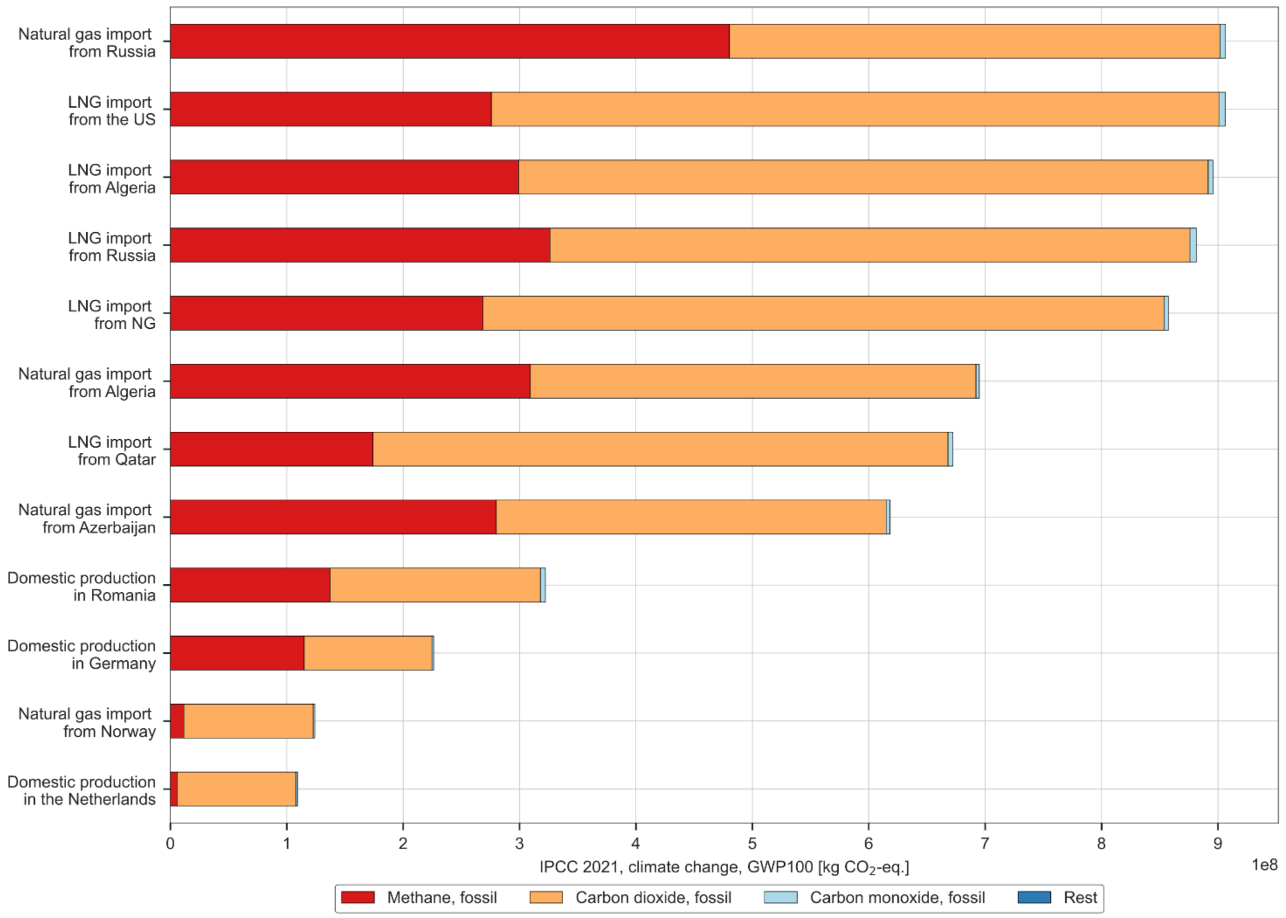

Figure S6. Life cycle carbon intensity of importing natural gas from the main suppliers of the EU in gaseous phase and as LNG broken down by elementary flow contribution, related to Figure 2. The functional unit considered is 1 bcm of natural gas imported in the gaseous phase in pipelines or as LNG followed by its regasification from the main suppliers to the EU. The LCIA method employed was the IPCC 2021, 100-year averaged GWP in kilogram of CO_2 equivalents (kg CO_2-eq) [S2].

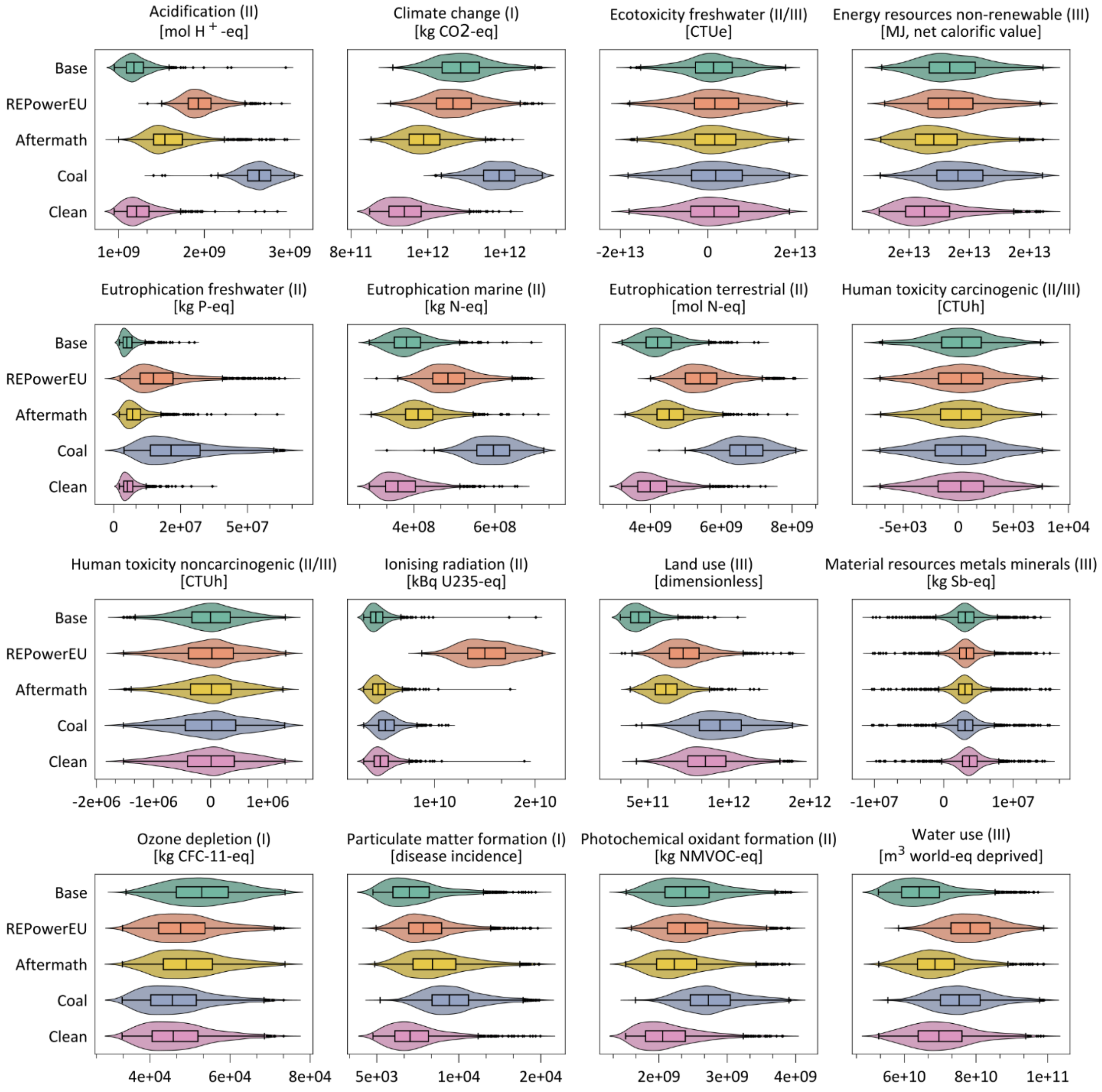

Figure S7. Violin plots for the 1000-iteration Monte Carlo simulation outcomes of the LCIA results of the natural gas supply and demand scenarios in the EU with the Environmental Footprint v3.1 methods, related to Figures 3, 4, and 5.

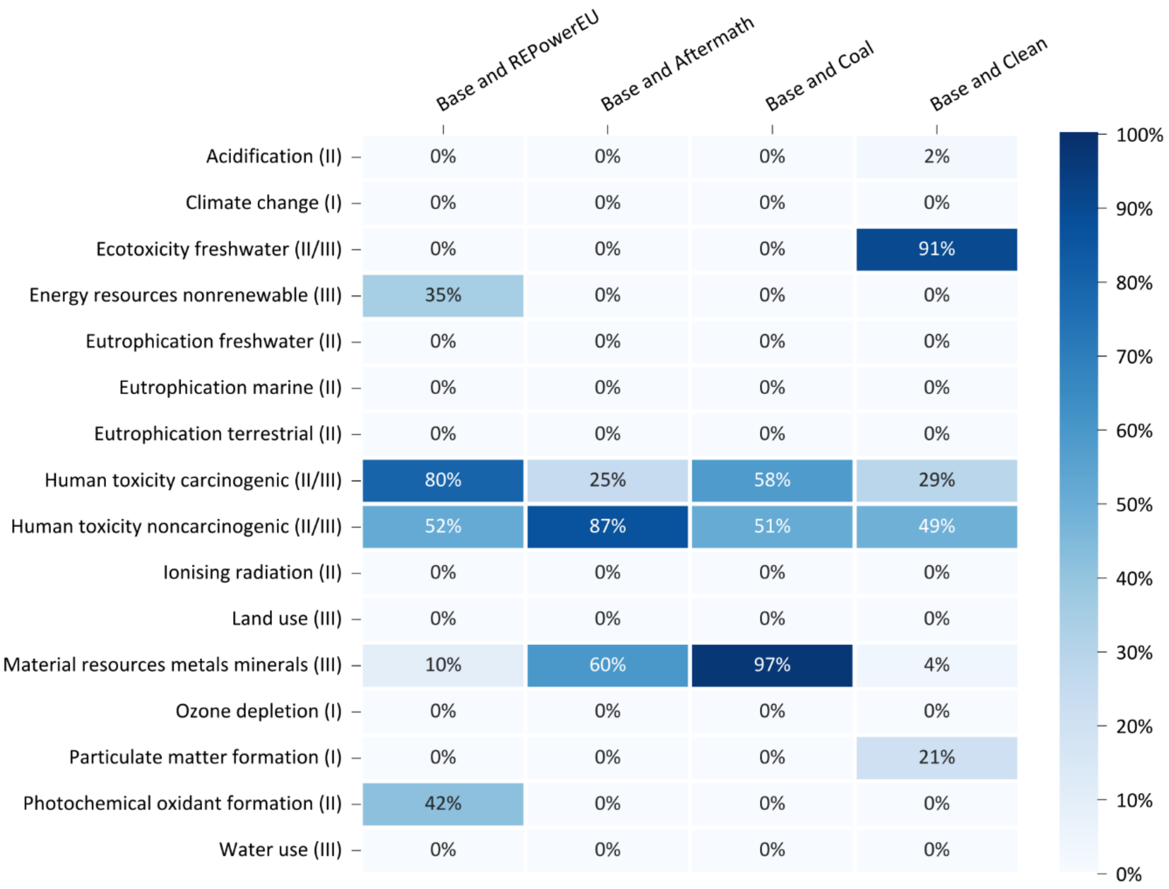

Figure S8. Scenario statistical significance based on the p-value of the paired sample t-test [S4], related to Figures 3 and 4. We check for the null hypothesis of equal means among the LCIA outcomes with the EF 3.1 methods for the Base and the alternative scenarios of natural gas supply and demand, considering the 1000-iteration Monte Carlo simulations. Values below 5% allow rejecting the null hypothesis, granting statistically significant distinction between the scenarios.

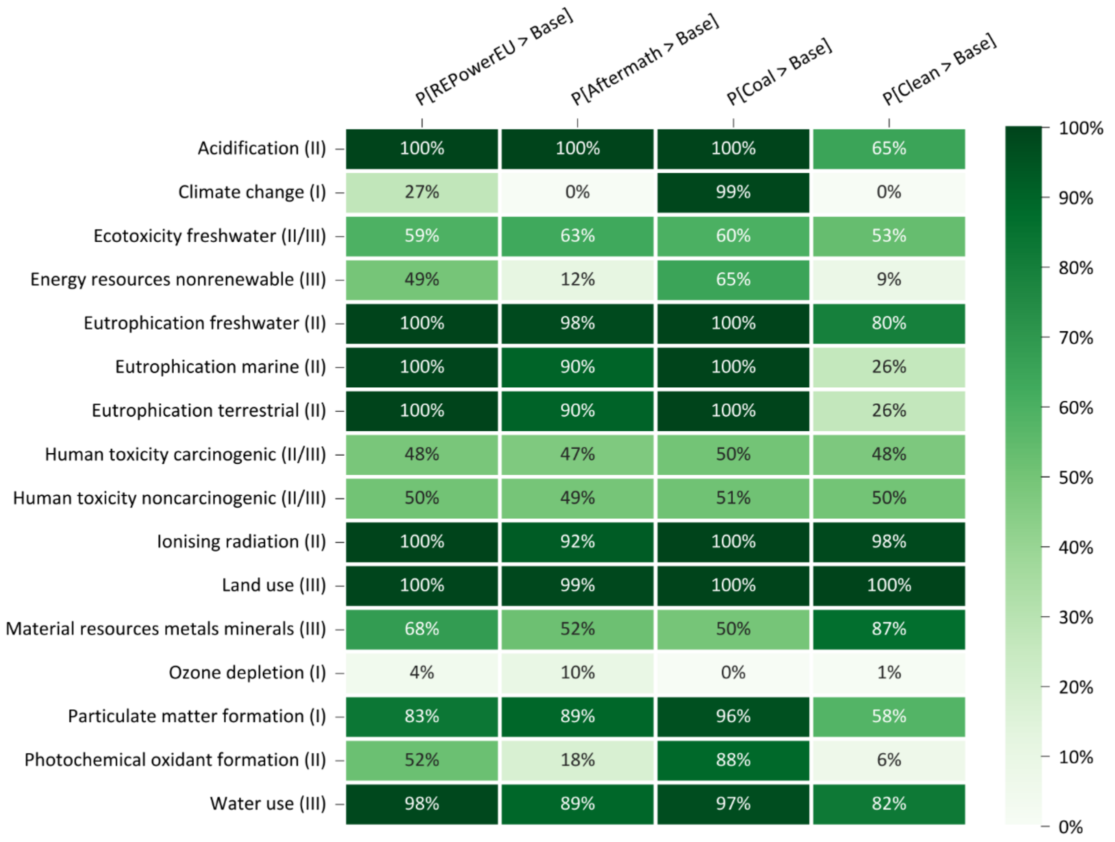

Figure S9. Probability that the LCIA results with the EF 3.1 methods of the alternative scenarios is higher than the Base scenario considering 1000-iteration Monte Carlo simulations, related to Figures 3 and 4. Considering 25% and 75% as the probability of increased emissions thresholds for the clear statistical comparison, we find an increased environmental burden in acidification, eutrophication (freshwater, marine, and terrestrial), ionising radiation, land use, particulate matter formation, and water use, while reducing the burden for ozone depletion, when comparing Base to the REPowerEU alternative scenario. Similar behaviour is observed when comparing it with the Coal scenario, except that we find a more prominent environmental impact increase in climate change, land use, and photochemical oxidant formation. The Clean scenario, on the other hand, provides undoubted emission reduction in climate change, non-renewable energy resources, land use, ozone depletion, and photochemical oxidant formation, while maintaining clear increased emission in freshwater eutrophication, ionising radiation, metal and mineral resources, and water use.

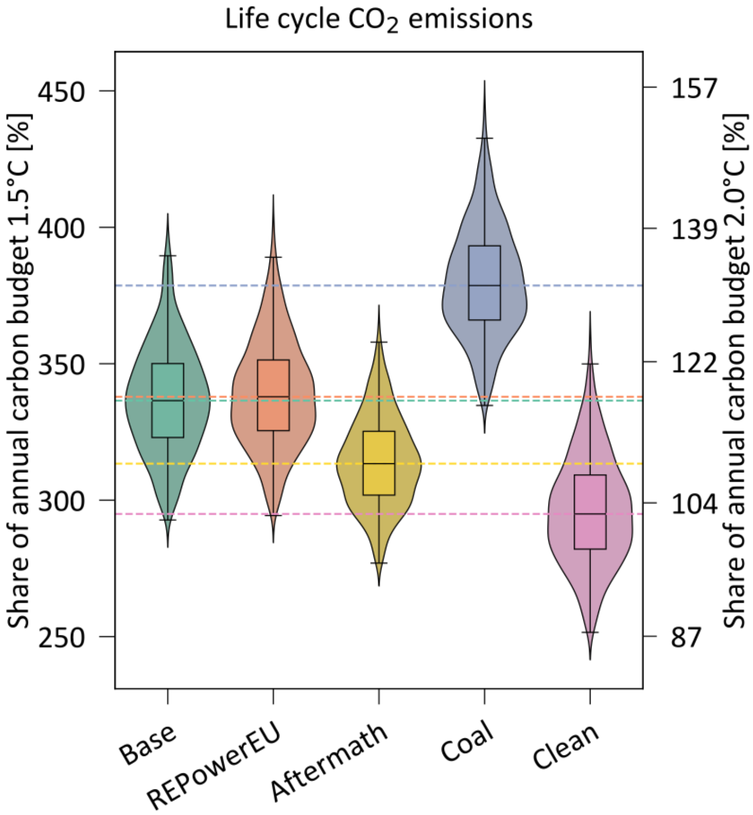

Figure S10. CO_2 emission budget transgression based on a constant yearly quota distributed by the world's population, related to Figure 2. We find that the results from the constant yearly per-capita budget [S5] in the main text are consistent with a fixed yearly carbon budget allocated by the population in each year. Notice that the latter approach results in a higher per-capita carbon budget for individuals today compared to future years, as the yearly carbon budget (the numerator in the equation) remains constant while the population (the denominator in the equation) increases over time. Mean transgression level results range from 277 and 359 % of the 1.5 °C budget and 96 and 125 % of the 2.0 °C budget across scenarios.

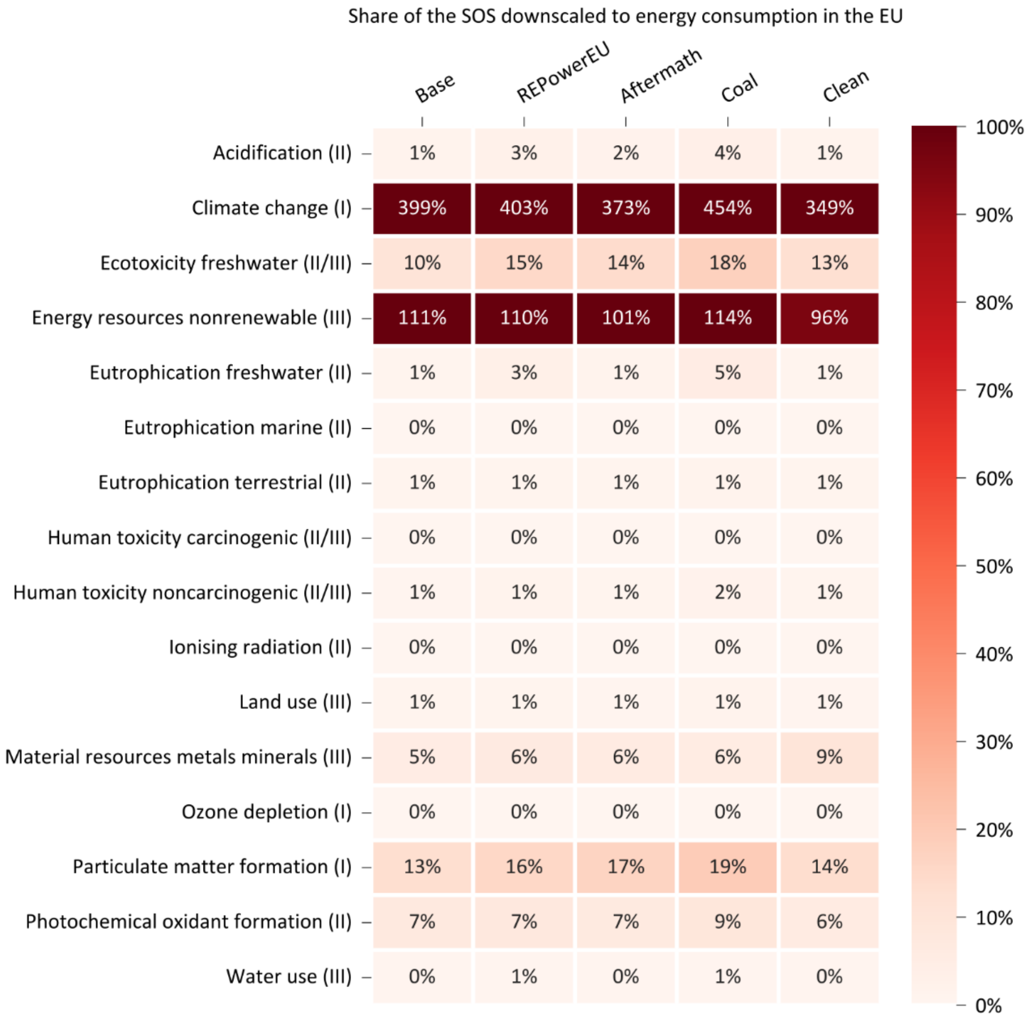

Figure S11. Planetary bounds allocation associated with the natural gas supply and demand scenarios due to the crisis in the EU, related to Figure 6. The thresholds defined for the EF impact categories [S6] derived from the Planetary Boundaries framework [S7, S8]. The share of the Earth's carrying capacity is downscaled to the EU's population based on the egalitarian principle [S5, S9]. The most relevant impact categories in terms of absolute sustainability for the EU's natural gas supply and energy-related demand are climate change, non-renewable energy resource use, particulate matter formation, freshwater ecotoxicity, and photochemical oxidant formation

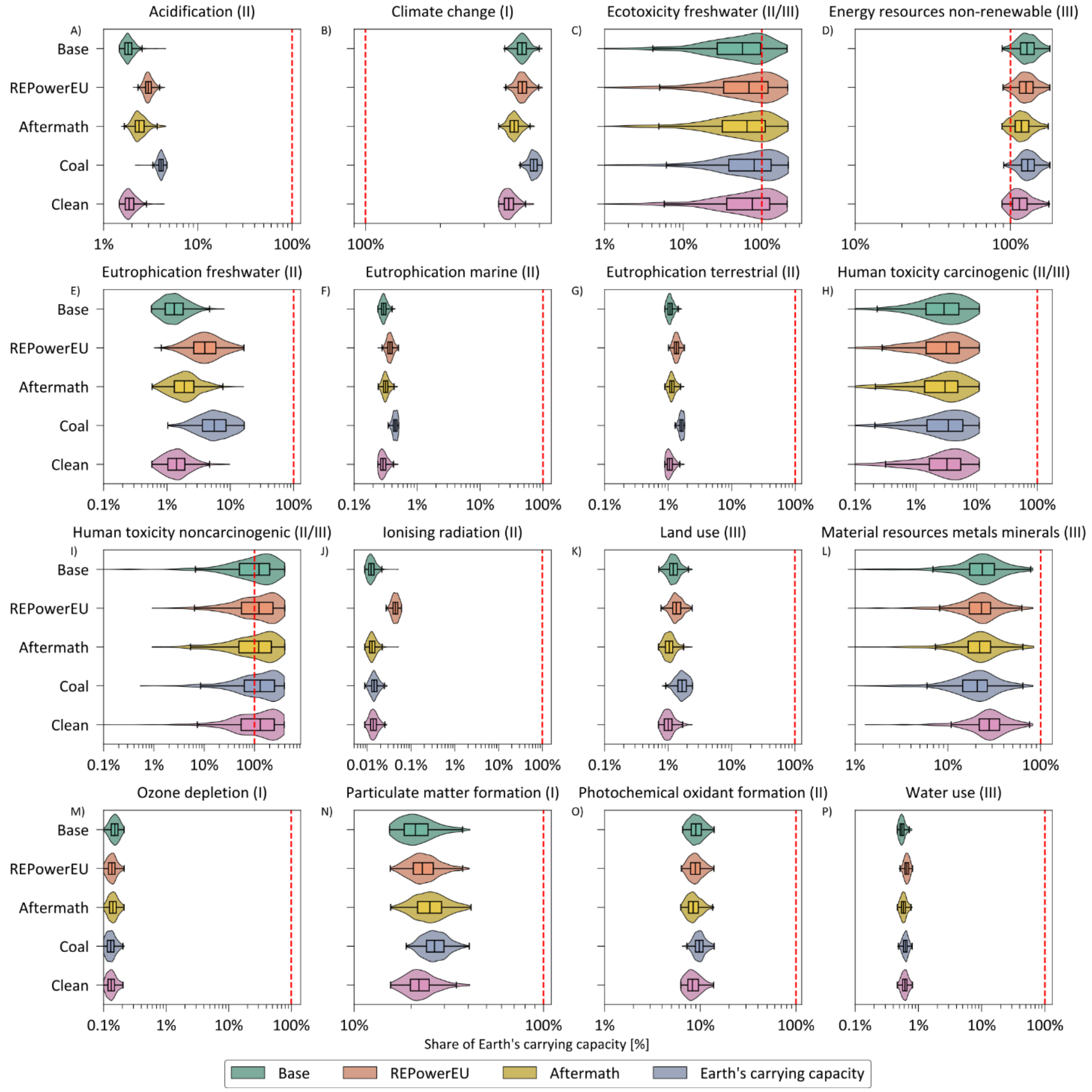

Figure S12. Planetary bounds allocation distribution associated with the natural gas supply and demand scenarios due to the crisis in the EU, related to Figure 6. Due to the inherent uncertainties in the life cycle inventory, some impact categories could exacerbate their nominal results**.** Nevertheless, the environmental impacts in those categories with a low share of the Earth's carrying capacity should not be overlooked because (i) the employed downscaling principle considers all anthropogenic emissions in the EU and (ii) the threshold for the Earth's carrying capacity involves potentially high uncertainty themselves [S10, S11].

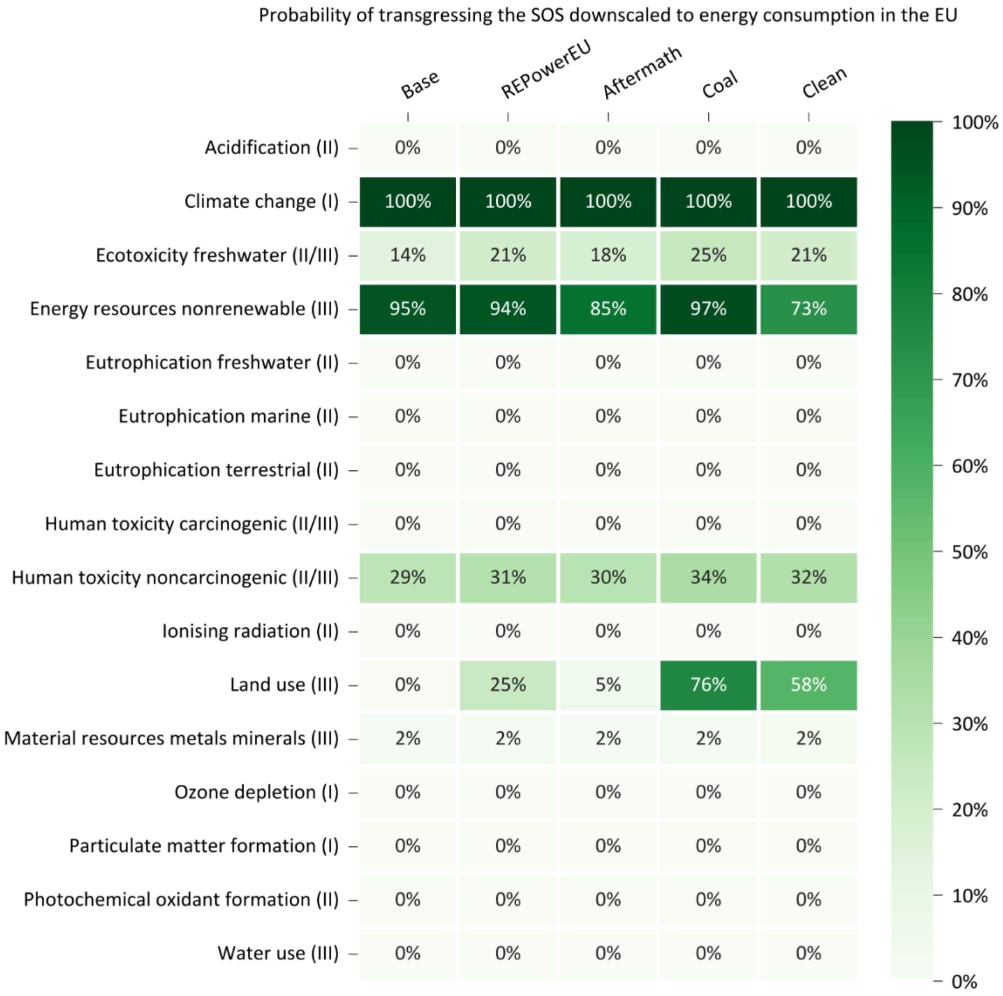

Figure S13. Probability of the natural gas supply and demand scenarios due to the crisis in the EU transgressing the Earth's ecological thresholds allocated to the EU's population, related to Figure 6. Non-renewable energy resource consumption presents a probability of transgressing the yearly Earth's carrying capacity downscaled to the EU population ranging from 73% to 97% for the Base, REPowerEU, and Coal scenarios.

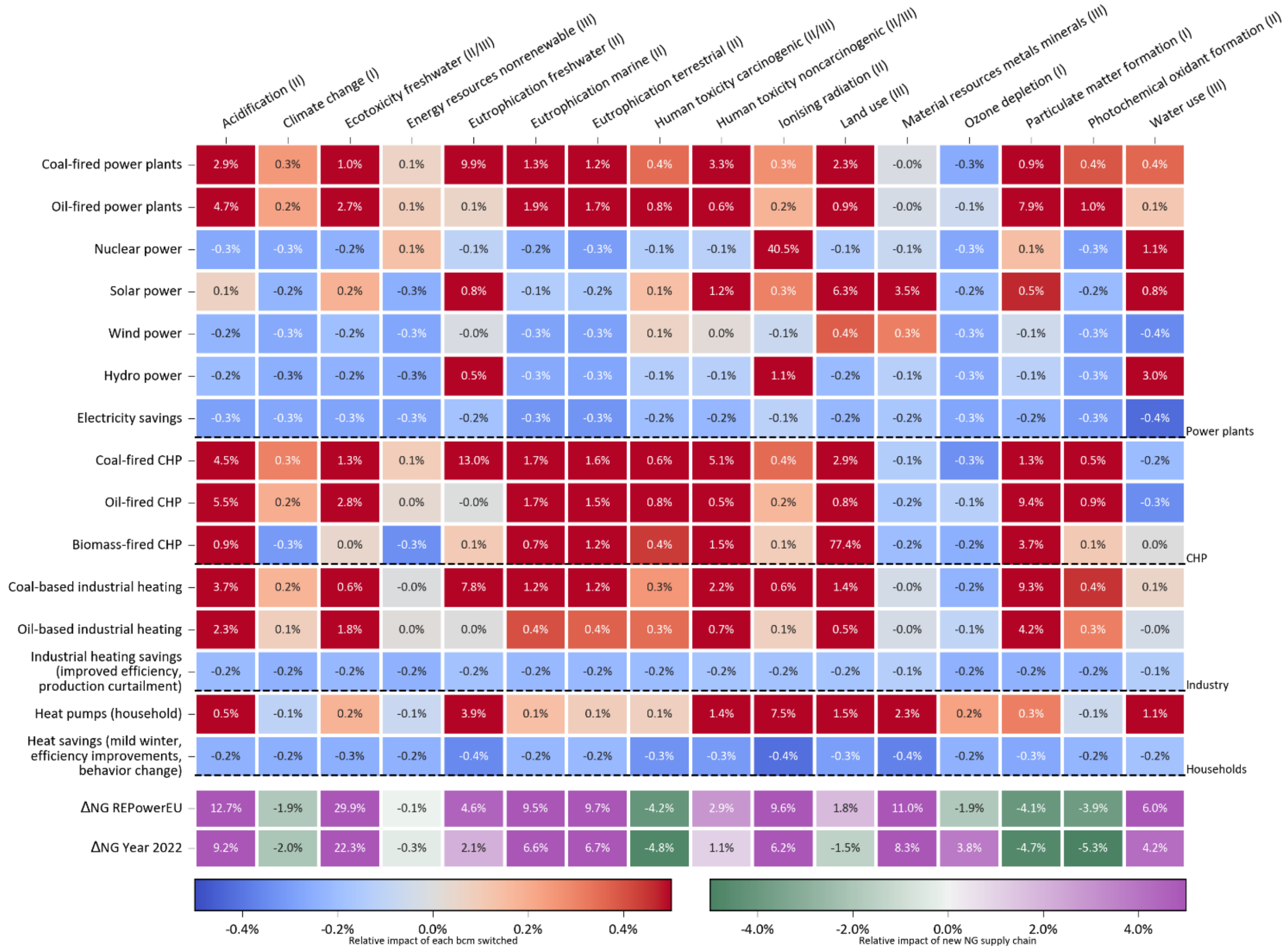

Figure S14. Relative change in life cycle environmental impacts from individual measures to reduce Russian gas consumption, related to Figure 7. The relative impacts on eutrophication (freshwater, marine, and terrestrial), human toxicity (carcinogenic and noncarcinogenic), ionising radiation, material resource consumption, ozone depletion, and water use range from −0.4 to 40.5%.

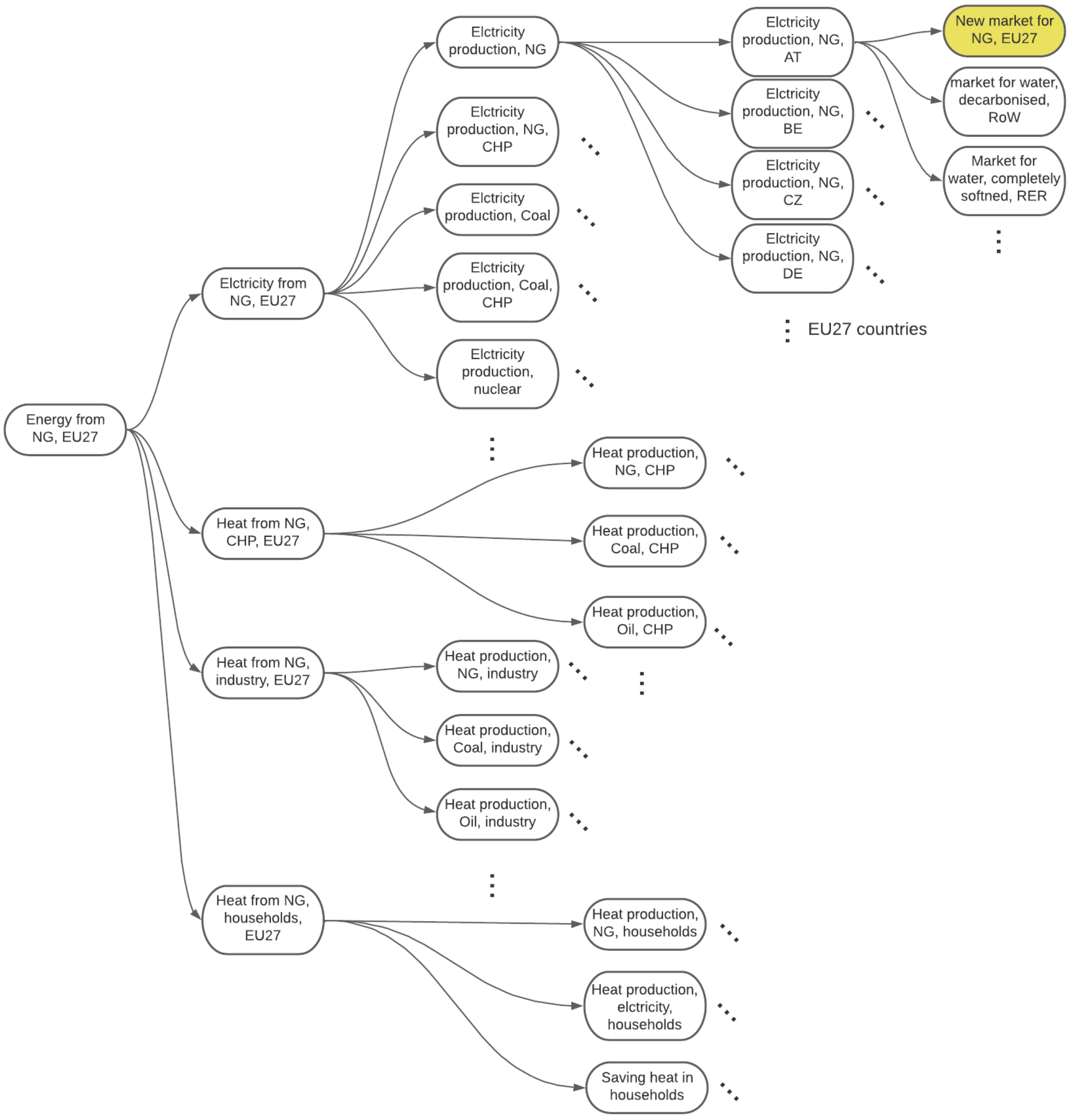

Figure S15. Schematic representation of the proposed life cycle inventory and system boundaries, related to STAR Methods. The functional unit comprises the energy (heat and electricity) production in the EU (originally) from natural gas and its replacements.

Supplemental Tables

Table S1. Natural gas supply composition for the Base, REPowerEU, and Aftermath scenarios [S12, S13], related to Figure 1.

	Supplier	Base [bcm]	REPowerEU [bcm]	Aftermath [bcm]
Domestic production [S14]	Denmark	1.3	1.3	1.3
	Germany	4.5	4.5	4.5
	Italy	3.2	3.2	3.2
	Netherlands	18.1	18.1	18.1
	Poland	3.9	3.9	3.9
	Romania	8.5	8.5	8.5
Pipeline imports [S15]	Norway	96.4	96.4	94.9
	Russia	127.6	17.6	30.0
	Algeria	35.2	35.2	33.7
	Azerbaijan	11.5	21.5	12.7
	Other Europe	16.9	16.9	26.7
	Libya	2.9	2.9	2.9
LNG imports [S15]	USA	38.6	88.6	61.0
	Qatar	17.1	17.1	21.4
	Russia	16.3	16.3	18.5
	Nigeria	15.9	15.9	28.2
	Algeria	8.6	8.6	5.7
Stock change [S15]		12.7	12.7	22.1
Total available NG		427	377	377

bcm: billion cubic meters. LNG: liquified natural gas.

Table S2. Natural gas demand breakdown for the pre-invasion (Base) and crisis management (REPowerEU and Aftermath), related to Figure 1.

Activity	EU natural gas demand [bcm] [S16]	Sector	Base [bcm]	REPowerEU [bcm]	Aftermath [bcm] [S17]
Electricity production	49.7	Transformation	49.7	18.7	51.7
Combined heat and power	62.1		62.1	62.1	62.1
Heat production	8.1		8.1	8.1	8.1
Oil and natural gas extraction plants	2.4	Energy	13.6	13.6	13.6
Petroleum refineries	10.5				
Other energy use in the energy sector	0.7				
Iron and steel	9.3	Industry – energy use	96.9	96.9	71.8
Chemical and petrochemical	23.0				
Non-ferrous metals	4.4				
Non-metallic minerals	16.2				
Transport equipment	3.0				
Machinery	7.0				
Mining and quarrying	1.0				
Food, beverages, tobacco	16.0				
Paper, pulp, and printing	8.9				
Wood and wood products	0.7				
Other energy use in industrial sectors	7.3				
Feedstock	17.1	Industry – non-energy use	N/A	N/A	N/A
Transport	4.5	Transport	N/A	N/A	N/A
Ambient, water heating	153.9	Household & service	153.9	134.9	125.4

N/A: Not accounted for in the scenarios because they remain unchanged in all scenarios and neglected for the alternative solutions.

Table S3. Natural gas demand conversion to final energy demand, related to Figure 1.

Activity	Natural gas demand [bcm]	Energy production [TWh]	Efficiency	Considerations
Power plants	1.0	5.0	51%	Eurostat data – Simplified balance for electricity and derived heat [S16]
CHP electricity	1.0	5.0	51%	
CHP heat		3.0	31%	
Heat in the Industry	1.0	9.0	92%	Average efficiency from ecoinvent 3.9.1 [S18]
Heat in households and the service sector	1.0	9.0	92%	

Table S4. Statistical results of a 1000-iteration Monte Carlo simulation of the LCIA results for the Base natural gas supply and demand scenario, related to Figure 5.

EF v3.1 [S19]	Baseline	Mean	Std	QCD [%]	Min	Q_1	Median	Q_3	Max
Acidification	8.93e+11	1.19e+09	1.52e+08	8.62	8.52e+08	1.08e+09	1.17e+09	1.28e+09	1.72e+09
Climate change	9.00e+08	1.09e+12	6.86e+10	4.37	9.40e+11	1.04e+12	1.08e+12	1.13e+12	1.28e+12
Ecotoxicity freshwater	1.00e+12	1.09e+12	6.40e+12	318.00	-1.88e+13	-2.89e+12	1.24e+12	5.55e+12	1.68e+13
Energy resources non-renewable	8.81e+11	1.88e+13	2.88e+12	10.30	1.30e+13	1.68e+13	1.84e+13	2.06e+13	2.78e+13
Eutrophication freshwater	1.60e+13	5.30e+06	2.68e+06	32.80	1.30e+06	3.35e+06	4.69e+06	6.61e+06	1.77e+07
Eutrophication marine	3.19e+06	3.85e+08	4.91e+07	8.43	2.82e+08	3.51e+08	3.80e+08	4.15e+08	5.60e+08
Eutrophication terrestrial	3.01e+08	4.25e+09	5.55e+08	8.40	3.01e+09	3.87e+09	4.19e+09	4.58e+09	6.19e+09
Human toxicity carcinogenic	3.29e+09	2.37e+02	2.95e+03	670.00	-8.28e+03	-1.56e+03	3.12e+02	2.10e+03	8.51e+03
Human toxicity noncarcinogenic	1.89e+02	-1.13e+04	5.29e+05	4,890.00	-1.65e+06	-3.30e+05	-3.86e+03	3.44e+05	1.35e+06
Ionising radiation	1.73e+03	4.27e+09	9.44e+08	14.70	2.52e+09	3.57e+09	4.12e+09	4.80e+09	7.63e+09
Land use	3.14e+09	4.47e+11	9.66e+10	14.50	2.46e+11	3.75e+11	4.35e+11	5.02e+11	7.93e+11
Material resources metals minerals	3.01e+11	3.21e+06	4.01e+06	36.10	-2.16e+07	2.06e+06	3.11e+06	4.40e+06	2.97e+07
Ozone depletion	7.25e+05	5.44e+04	9.71e+03	13.10	3.62e+04	4.69e+04	5.35e+04	6.11e+04	8.25e+04
Particulate matter formation	4.48e+04	7.27e+03	2.03e+03	16.70	3.94e+03	5.82e+03	6.90e+03	8.16e+03	1.63e+04
Photochemical oxidant formation	4.42e+03	2.48e+09	5.20e+08	13.60	1.62e+09	2.10e+09	2.41e+09	2.77e+09	4.37e+09
Water use	1.94e+09	6.47e+10	8.74e+09	8.69	4.80e+10	5.83e+10	6.35e+10	6.94e+10	9.40e+10
Land use – LANCA v2.5 [S20]	6.85e+09	1.02e+10	2.37e+09	14.60	5.77e+09	8.52e+09	9.81e+09	1.14e+10	1.94e+10
IPCC 2021 – GWP100a [S2]	1.00e+12	1.09e+12	6.88e+10	4.43	9.43e+11	1.04e+12	1.09e+12	1.13e+12	1.29e+12
IPCC 2021 – GWP20a [S2]	1.19e+12	1.32e+12	1.09e+11	5.76	1.12e+12	1.24e+12	1.30e+12	1.39e+12	1.70e+12
Life-cycle CO_2 emission	8.93e11	9.54e+11	5.59e+10	4.03	8.28e+11	9.14e+11	9.52e+11	9.90e+11	1.12e+12

Table S5. Statistical results of a 1000-iteration Monte Carlo simulation of the LCIA results for the REPowerEU natural gas supply and demand scenario, related to Figure 5.

EF v3.1 [S19]	Baseline	Mean	Std	QCD [%]	Min	Q_1	Median	Q_3	Max
Acidification	1.63e+09	1.96e+09	2.00e+08	6.71	1.55e+09	1.82e+09	1.93e+09	2.08e+09	2.70e+09
Climate change	9.90e+11	1.07e+12	6.53e+10	4.14	9.24e+11	1.02e+12	1.06e+12	1.11e+12	1.25e+12
Ecotoxicity freshwater	1.28e+12	1.57e+12	7.94e+12	279.00	-2.15e+13	-3.39e+12	1.63e+12	7.16e+12	2.03e+13
Energy resources non-renewable	1.59e+13	1.88e+13	2.94e+12	10.20	1.33e+13	1.66e+13	1.84e+13	2.04e+13	2.84e+13
Eutrophication freshwater	1.19e+07	1.80e+07	1.15e+07	38.90	3.97e+06	9.98e+06	1.49e+07	2.27e+07	6.87e+07
Eutrophication marine	3.99e+08	4.92e+08	6.11e+07	8.09	3.72e+08	4.47e+08	4.86e+08	5.26e+08	7.24e+08
Eutrophication terrestrial	4.43e+09	5.50e+09	6.92e+08	8.07	4.16e+09	5.00e+09	5.40e+09	5.87e+09	8.05e+09
Human toxicity carcinogenic	1.91e+02	2.22e+02	3.07e+03	941.00	-8.22e+03	-1.83e+03	2.70e+02	2.26e+03	8.51e+03
Human toxicity noncarcinogenic	3.30e+03	-8.03e+03	6.07e+05	5,110.00	-1.79e+06	-3.92e+05	2.11e+04	4.08e+05	1.51e+06
Ionising radiation	1.32e+10	1.59e+10	3.26e+09	13.60	1.00e+10	1.35e+10	1.54e+10	1.78e+10	2.80e+10
Land use	4.96e+11	7.35e+11	1.41e+11	12.50	4.61e+11	6.34e+11	7.18e+11	8.15e+11	1.37e+12
Material resources metals minerals	9.09e+05	3.32e+06	3.59e+06	31.40	-1.64e+07	2.26e+06	3.24e+06	4.33e+06	2.90e+07
Ozone depletion	4.01e+04	4.86e+04	8.56e+03	12.20	3.11e+04	4.21e+04	4.77e+04	5.38e+04	7.41e+04
Particulate matter formation	5.20e+03	8.11e+03	1.64e+03	12.60	5.18e+03	6.95e+03	7.84e+03	8.95e+03	1.44e+04
Photochemical oxidant formation	1.96e+09	2.47e+09	4.71e+08	12.30	1.63e+09	2.13e+09	2.39e+09	2.73e+09	4.17e+09
Water use	6.44e+10	7.97e+10	8.80e+09	7.26	6.21e+10	7.33e+10	7.88e+10	8.48e+10	1.11e+11
Land use – LANCA v2.5 [S20]	7.68e+09	1.19e+10	2.73e+09	14.80	6.77e+09	9.96e+09	1.14e+10	1.34e+10	2.32e+10
IPCC 2021 – GWP100a [S2]	9.92e+11	1.07e+12	6.56e+10	4.16	9.27e+11	1.02e+12	1.07e+12	1.11e+12	1.26e+12
IPCC 2021 – GWP20a [S2]	1.14e+12	1.25e+12	9.47e+10	5.20	1.06e+12	1.18e+12	1.24e+12	1.31e+12	1.53e+12
Life-cycle CO_2 emission	9.03e+11	9.60e+11	5.55e+10	3.83	8.33e+11	9.21e+11	9.56e+11	9.94e+11	1.14e+12

Table S6. Statistical results of a 1000-iteration Monte Carlo simulation of the LCIA results for the Aftermath natural gas supply and demand scenario, related to Figure 5.

EF v3.1 [S19]	Baseline	Mean	Std	QCD [%]	Min	Q_1	Median	Q_3	Max
Acidification	1.23e+09	1.62e+09	3.30e+08	10.90	1.12e+09	1.41e+09	1.55e+09	1.75e+09	3.27e+09
Climate change	9.16e+11	9.89e+11	5.62e+10	3.96	8.65e+11	9.49e+11	9.86e+11	1.03e+12	1.14e+12
Ecotoxicity freshwater	1.17e+12	1.44e+12	7.15e+12	268.00	-1.97e+13	-2.92e+12	1.55e+12	6.40e+12	1.83e+13
Energy resources non-renewable	1.47e+13	1.73e+13	2.64e+12	9.92	1.21e+13	1.55e+13	1.70e+13	1.89e+13	2.53e+13
Eutrophication freshwater	5.14e+06	8.05e+06	4.39e+06	33.70	2.11e+06	4.89e+06	7.02e+06	9.87e+06	2.73e+07
Eutrophication marine	3.30e+08	4.17e+08	5.26e+07	8.03	3.14e+08	3.80e+08	4.11e+08	4.47e+08	6.15e+08
Eutrophication terrestrial	3.62e+09	4.61e+09	5.91e+08	8.09	3.46e+09	4.19e+09	4.53e+09	4.93e+09	6.82e+09
Human toxicity carcinogenic	1.75e+02	2.03e+02	2.86e+03	796.00	-7.52e+03	-1.64e+03	2.49e+02	2.11e+03	7.90e+03
Human toxicity noncarcinogenic	2.19e+03	-9.37e+03	5.54e+05	16,000.00	-1.61e+06	-3.64e+05	1.37e+04	3.59e+05	1.38e+06
Ionising radiation	3.37e+09	4.53e+09	9.87e+08	14.10	2.68e+09	3.82e+09	4.36e+09	5.08e+09	8.05e+09
Land use	4.45e+11	6.17e+11	9.97e+10	10.70	4.08e+11	5.46e+11	6.11e+11	6.78e+11	9.45e+11
Material resources metals minerals	8.85e+05	3.13e+06	3.49e+06	32.00	-1.66e+07	2.11e+06	3.05e+06	4.09e+06	2.82e+07
Ozone depletion	4.17e+04	5.04e+04	8.94e+03	12.40	3.40e+04	4.35e+04	4.92e+04	5.58e+04	7.68e+04
Particulate matter formation	5.61e+03	8.97e+03	2.38e+03	15.60	5.21e+03	7.26e+03	8.47e+03	9.96e+03	1.98e+04
Photochemical oxidant formation	1.80e+09	2.30e+09	4.44e+08	12.90	1.52e+09	1.97e+09	2.23e+09	2.55e+09	3.84e+09
Water use	5.49e+10	6.94e+10	7.91e+09	7.40	5.33e+10	6.37e+10	6.85e+10	7.39e+10	9.58e+10
Land use – LANCA v2.5 [S20]	5.70e+09	8.73e+09	1.79e+09	13.40	5.05e+09	7.43e+09	8.60e+09	9.73e+09	1.51e+10
IPCC 2021 – GWP100a [S2]	9.18e+11	9.92e+11	5.64e+10	3.94	8.68e+11	9.52e+11	9.89e+11	1.03e+12	1.15e+12
IPCC 2021 – GWP20a [S2]	1.06e+12	1.16e+12	8.54e+10	4.99	9.96e+11	1.10e+12	1.15e+12	1.22e+12	1.43e+12
Life-cycle CO_2 emission	8.34e+11	8.89e+11	4.67e+10	3.73	7.83e+11	8.54e+11	8.87e+11	9.20e+11	1.03e+12

Table S7. Statistical results of a 1000-iteration Monte Carlo simulation of the LCIA results for the Coal natural gas supply and demand scenario, related to Figure 5.

EF v3.1 [S19]	Baseline	Mean	Std	QCD [%]	Min	Q_1	Median	Q_3	Max
Acidification	2.30e+09	2.68e+09	2.19e+08	5.42	2.23e+09	2.52e+09	2.66e+09	2.81e+09	3.44e+09
Climate change	1.11e+12	1.19e+12	6.68e+10	3.77	1.05e+12	1.15e+12	1.19e+12	1.24e+12	1.40e+12
Ecotoxicity freshwater	1.52e+12	1.87e+12	9.32e+12	288.00	-2.48e+13	-4.10e+12	1.91e+12	8.46e+12	2.34e+13
Energy resources non-renewable	1.65e+13	1.96e+13	2.94e+12	10.10	1.39e+13	1.74e+13	1.92e+13	2.13e+13	2.91e+13
Eutrophication freshwater	1.96e+07	2.95e+07	2.15e+07	43.90	5.20e+06	1.44e+07	2.28e+07	3.71e+07	1.28e+08
Eutrophication marine	5.02e+08	6.08e+08	6.67e+07	7.40	4.72e+08	5.59e+08	6.02e+08	6.48e+08	8.50e+08
Eutrophication terrestrial	5.62e+09	6.84e+09	7.70e+08	7.37	5.27e+09	6.29e+09	6.76e+09	7.29e+09	9.61e+09
Human toxicity carcinogenic	2.16e+02	2.53e+02	3.50e+03	1,210.00	-9.24e+03	-2.21e+03	3.17e+02	2.60e+03	9.42e+03
Human toxicity noncarcinogenic	4.66e+03	-6.37e+03	7.04e+05	5,240.00	-2.07e+06	-4.61e+05	2.27e+04	4.79e+05	1.66e+06
Ionising radiation	3.88e+09	5.31e+09	1.17e+09	14.30	3.06e+09	4.47e+09	5.11e+09	5.96e+09	9.16e+09
Land use	6.46e+11	9.99e+11	2.30e+11	14.10	5.76e+11	8.35e+11	9.60e+11	1.11e+12	1.90e+12
Material resources metals minerals	7.84e+05	3.15e+06	3.81e+06	35.20	-1.77e+07	2.02e+06	3.07e+06	4.21e+06	3.10e+07
Ozone depletion	3.81e+04	4.62e+04	8.51e+03	12.80	2.93e+04	3.97e+04	4.52e+04	5.14e+04	7.09e+04
Particulate matter formation	6.41e+03	9.75e+03	1.84e+03	12.00	6.44e+03	8.41e+03	9.46e+03	1.07e+04	1.66e+04
Photochemical oxidant formation	2.26e+09	2.82e+09	4.71e+08	10.60	1.96e+09	2.48e+09	2.75e+09	3.07e+09	4.48e+09
Water use	6.12e+10	7.65e+10	8.67e+09	7.42	5.90e+10	7.04e+10	7.57e+10	8.16e+10	1.07e+11
Land use – LANCA v2.5 [S20]	1.01e+10	1.56e+10	4.31e+09	17.70	8.15e+09	1.25e+10	1.48e+10	1.79e+10	3.56e+10
IPCC 2021 – GWP100a [S2]	1.11e+12	1.20e+12	6.71e+10	3.76	1.05e+12	1.15e+12	1.19e+12	1.24e+12	1.40e+12
IPCC 2021 – GWP20a [S2]	1.28e+12	1.40e+12	9.82e+10	4.73	1.20e+12	1.33e+12	1.39e+12	1.46e+12	1.68e+12
Life-cycle CO_2 emission	1.02e+12	1.08e+12	5.66e+10	3.58	9.47e+11	1.04e+12	1.07e+12	1.11e+12	1.26e+12

Table S8. Statistical results of a 1000-iteration Monte Carlo simulation of the LCIA results for the Clean natural gas supply and demand scenario, related to Figure 5.

EF v3.1 [S19]	Baseline	Mean	Std	QCD [%]	Min	Q_1	Median	Q_3	Max
Acidification	9.39e+08	1.23e+09	1.94e+08	10.50	8.41e+08	1.09e+09	1.20e+09	1.34e+09	1.97e+09
Climate change	8.58e+11	9.30e+11	6.53e+10	4.84	7.88e+11	8.83e+11	9.27e+11	9.73e+11	1.12e+12
Ecotoxicity freshwater	1.07e+12	1.27e+12	8.47e+12	379.00	-2.37e+13	-4.21e+12	1.44e+12	7.22e+12	2.13e+13
Energy resources non-renewable	1.38e+13	1.64e+13	2.89e+12	11.40	1.11e+13	1.44e+13	1.61e+13	1.81e+13	2.57e+13
Eutrophication freshwater	3.64e+06	5.68e+06	2.80e+06	31.80	1.56e+06	3.62e+06	5.07e+06	7.00e+06	1.77e+07
Eutrophication marine	2.88e+08	3.67e+08	5.86e+07	10.30	2.58e+08	3.25e+08	3.58e+08	4.00e+08	5.97e+08
Eutrophication terrestrial	3.15e+09	4.05e+09	6.59e+08	10.40	2.82e+09	3.58e+09	3.94e+09	4.41e+09	6.62e+09
Human toxicity carcinogenic	1.86e+02	2.05e+02	3.25e+03	971.00	-8.41e+03	-1.91e+03	2.31e+02	2.35e+03	8.48e+03
Human toxicity noncarcinogenic	2.14e+03	-1.10e+04	6.46e+05	11,900.00	-1.83e+06	-4.20e+05	8.17e+03	4.27e+05	1.57e+06
Ionising radiation	3.52e+09	4.76e+09	1.08e+09	14.90	2.80e+09	3.98e+09	4.59e+09	5.38e+09	8.59e+09
Land use	7.02e+11	8.78e+11	1.77e+11	13.60	5.37e+11	7.50e+11	8.57e+11	9.86e+11	1.54e+12
Material resources metals minerals	1.34e+06	3.79e+06	3.78e+06	29.40	-1.73e+07	2.65e+06	3.70e+06	4.86e+06	3.10e+07
Ozone depletion	3.85e+04	4.66e+04	8.51e+03	12.60	2.99e+04	4.02e+04	4.57e+04	5.18e+04	7.12e+04
Particulate matter formation	4.52e+03	7.31e+03	1.75e+03	14.50	4.37e+03	6.07e+03	6.97e+03	8.12e+03	1.50e+04
Photochemical oxidant formation	1.62e+09	2.09e+09	4.63e+08	14.60	1.30e+09	1.75e+09	2.02e+09	2.35e+09	3.72e+09
Water use	5.47e+10	7.04e+10	9.35e+09	8.87	5.02e+10	6.37e+10	6.96e+10	7.60e+10	1.00e+11
Land use – LANCA v2.5 [S20]	4.95e+09	7.89e+09	1.95e+09	15.00	3.79e+09	6.54e+09	7.66e+09	8.86e+09	1.52e+10
IPCC 2021 – GWP100a [S2]	8.60e+11	9.34e+11	6.56e+10	4.85	7.91e+11	8.86e+11	9.30e+11	9.76e+11	1.12e+12
IPCC 2021 – GWP20a [S2]	9.89e+11	1.09e+12	9.31e+10	5.98	9.07e+11	1.02e+12	1.08e+12	1.15e+12	1.37e+12
Life-cycle CO_2 emission	7.83e+11	8.38e+11	5.62e+10	4.59	7.12e+11	7.98e+11	8.34e+11	8.75e+11	1.02e+12

Table S9. Optimal arrays of strategies to reduce the EU's natural gas consumption at different reduction targets, related to STAR Methods. They focus on the absolute environmental implications (i.e., on the ecological limits) of strategies to reduce natural gas consumption in EU countries beyond GHG emissions only to support the development of long-term strategies to reduce natural gas demand.

**Optimal array of strategies (bcm replaced)**					
Biomass-fired CHP	0	0	70.2	70.2	70.2
Coal-based industrial heating	0	0	0	0	0
Coal-fired CHP	0	0	0	0	0
Coal-fired power plants	0	0	0	0	0
Electricity savings	0	0	0	0	0
Heat pumps (household)	0	0	29.8	153.9	153.9
Heat savings (household)	0	0	0	0	0
Hydropower	0	0	0	0	0
Industrial heating savings	0	0	0	0	0
Nuclear power	0	0	0	0	0
Oil-based industrial heating	0	0	0	109.9	0
Oil-fired CHP	0	0	0	0	0
Oil-fired power plants	0	0	0	0	0
Solar power	0	0	0	0	0
Wind power	0	50	50	50	50
**Optimisation**					
Demand reduction (bcm)	0.0	50.0	150.0	384.0	Free
Objective value (mean absolute transgression)	38.0%	30.1%	25.2%	32.4%	24.3%
**Impact breakdown**					
Acidification (II)	1.4%	1.4%	2.4%	6.8%	3.4%
Climate change (I)	398.9%	346.0%	243.0%	219.0%	180.0%
Ecotoxicity freshwater (II/III)	10.4%	11.7%	12.6%	34.9%	14.9%
Energy resources non-renewable (III)	110.6%	94.8%	65.9%	57.3%	56.1%
Eutrophication freshwater (II)	0.8%	0.9%	9.7%	44.0%	44.0%
Eutrophication marine (II)	0.2%	0.2%	0.3%	0.5%	0.4%
Eutrophication terrestrial (II)	0.8%	0.8%	1.5%	2.0%	1.6%
Human toxicity carcinogenic (II/III)	0.3%	0.3%	0.4%	0.5%	0.4%
Human toxicity noncarcinogenic (II/III)	0.7%	0.7%	1.6%	3.2%	2.7%
Ionising radiation (II)	0.0%	0.0%	0.1%	0.3%	0.3%
Land use (III)	0.8%	0.6%	0.5%	0.3%	0.4%
Material resources metals minerals (III)	5.1%	6.3%	9.1%	23.7%	23.8%
Ozone depletion (I)	0.1%	0.1%	0.1%	0.1%	0.1%
Particulate matter formation (I)	13.3%	12.3%	48.6%	116.0%	53.8%
Photochemical oxidant formation (II)	7.4%	5.9%	6.4%	8.3%	5.8%
Water use (III)	0.4%	0.4%	0.5%	1.1%	1.1%

Table S10. Life cycle inventory for the functional unit that considers the energy use of natural gas in the EU countries across scenarios, related to STAR Methods.

All scenarios	Unit	Product	Activity	Location
**Reference product**				
1.000	kilowatt hour	Reference product	energy from NG, overall mix	EU27
**Technosphere flows**				
0.1991	kilowatt hour	electricity, high voltage	electricity from NG	EU27
0.2823	megajoule	heat, district or industrial	heat from NG, CHP	EU27
1.0315	megajoule	heat, district or industrial	heat from NG, industry	EU27
1.5693	megajoule	heat, central or small-scale	heat from NG, households	EU27

Table S11. Life cycle inventory for the electricity production from natural gas in the EU countries across scenarios, related to STAR Methods.

Base	REPowerEU	Aftermath	Coal	Clean	Unit	Product	Activity	Location
**Reference product**								
1.000	1.000	1.000	1.000	1.000	kilowatt hour	Electricity from NG	Electricity production from NG	EU27
**Technosphere flows**								
0.4442	0.1669	0.4621	0.0	0.0864	kilowatt hour	electricity, high voltage	electricity production, NG	EU27
0.5558	0.5558	0.5558	0.5558	0.5558			electricity production, NG, CHP	
0.0	0.2147	0.0537	0.4442	0.0			electricity production, coal	
0.0	0.0	0.0	0.0	0.0			electricity production, coal, CHP	
0.0	0.0626	0.0	0.0	0.0			electricity production, nuclear	
0.0	0.0	0.0492	0.0	0.1789			electricity production, wind	
0.0	0.0	0.0492	0.0	0.1789			electricity production, photovoltaic	
0.0	0.0	0.0	0.0	0.0			electricity production, biomass, CHP	
0.0	0.0	0.0	0.0	0.0			electricity production, oil	
0.0	0.0	0.0	0.0	0.0			electricity production, oil, CHP	
0.0	0.0	0.0	0.0	0.0			electricity production, hydropower	
0.0	0.0	0.1342	0.0	0.0894			electricity savings	

Table S12. Life cycle inventory for heat production in CHP from natural gas in the EU countries across scenarios, related to STAR Methods.

Base	REPowerEU	Aftermath	Coal	Clean	Unit	Product	Activity	Location
**Reference product**								
1.000	1.000	1.000	1.000	1.000	megajoule	Heat from NG, CHP	Heat production from NG, CHP	EU27
**Technosphere flows**								
1.000	1.000	1.000	1.000	1.000	megajoule	heat, district or industrial	heat production from NG, CHP	EU27
0.0	0.0	0.0	0.0	0.0			heat production from coal, CHP	
0.0	0.0	0.0	0.0	0.0			heat production from biomass, CHP	
0.0	0.0	0.0	0.0	0.0			heat production from oil, CHP	

Table S13. Life cycle inventory for heat production in the industry from natural gas in the EU countries across scenarios, related to STAR Methods.

Base	REPowerEU	Aftermath	Coal	Clean	Unit	Product	Activity	Location
**Reference product**								
1.000	1.000	1.000	1.000	1.000	megajoule	Heat from NG, industry	Heat production from NG, industry	EU27
**Technosphere flows**								
1.000	1.000	0.7736	1.000	1.000	megajoule	heat, district or industrial, natural gas	heat production, natural gas, at industrial furnace >100kW	EU27
0.0	0.0	0.0	0.0	0.0		heat, district or industrial, other than natural gas	heat production, at hard coal industrial furnace 1-10MW	
0.0	0.0	0.0634	0.0	0.0			heat production, heavy fuel oil, at industrial furnace 1MW	
0.0	0.0	0.0272	0.0	0.0			heat industry, savings, efficiency improvements	
0.0	0.0	0.1358	0.0	0.0			heat industry, savings, production curtailment	

Table S14. Life cycle inventory for heat production in households and the service sector from natural gas in the EU countries across scenarios, related to STAR Methods.

Base	REPowerEU	Aftermath	Coal	Clean	Unit	Product	Activity	Location
**Reference product**								
1.000	1.000	1.000	1.000	1.000	megajoule	Heat from NG, households	Heat production from NG, households	EU27
**Technosphere flows**								
1.000	0.8765	0.8148	1.000	0.9350	megajoule	heat, central or small-scale	heat production from NG, central or small-scale	EU27
0.0	0.0585	0.0091	0.0	0.0			heat production from electricity	
0.0	0.0	0.1170	0.0	0.0			heat savings in households, mild weather	
0.0	0.0	0.0227	0.0	0.0			heat savings in households, efficiency improvements	
0.0	0.0650	0.0364	0.0	0.0650			heat savings in households, behaviour change	

Table S15. Life cycle inventory for the natural gas supply mix across scenarios, related to STAR Methods.

Base [S14]	REPowerEU [S12,S14]	Aftermath	Coal	Clean	Unit	Product	Activity	Location
**Reference product**								
1.000	1.000	1.000	1.000	1.000	Cubic meter	Natural gas, high pressure	Market group for natural gas, high pressure	EU27
**Technosphere flows**								
1.0000	1.0000	1.000	1.0000	1.0000	cubic meter	natural gas distribution within EU27	natural gas distribution within EU27	EU27
0.0010	0.0010	0.0010	0.0010	0.0010	cubic meter	natural gas, high pressure	market group for natural gas, high pressure	EU27
0.0134	0.0152	0.0152	0.0152	0.0152	cubic meter	natural gas, high pressure	natural gas, high pressure, domestic production in DE	EU27
0.0539	0.0610	0.0610	0.0610	0.0610	cubic meter	natural gas, high pressure	natural gas, high pressure, domestic production in NL	EU27
0.0253	0.0287	0.0286	0.0287	0.0287	cubic meter	natural gas, high pressure	natural gas, high pressure, domestic production in RO	EU27
0.0290	0.0638	0.0396	0.0638	0.0638	cubic meter	natural gas, high pressure	natural gas, high pressure, import from AZ	EU27
0.0886	0.1044	0.1050	0.1044	0.1044	cubic meter	natural gas, high pressure	natural gas, high pressure, import from DZ	EU27
0.2425	0.2857	0.2954	0.2857	0.2857	cubic meter	natural gas, high pressure	natural gas, high pressure, import from NO	EU27
0.3210	0.0523	0.0933	0.0523	0.0523	cubic meter	natural gas, high pressure	natural gas, high pressure, import from RU	EU27
0.0202	0.0228	0.0152	0.0228	0.0228	cubic meter	natural gas, high pressure	regasification LNG from DZ	EU27
0.0373	0.0423	0.0756	0.0423	0.0423	cubic meter	natural gas, high pressure	regasification LNG from NG	EU27
0.0402	0.0455	0.0575	0.0455	0.0455	cubic meter	natural gas, high pressure	regasification LNG from QA	EU27
0.0382	0.0433	0.0498	0.0433	0.0433	cubic meter	natural gas, high pressure	regasification LNG from RU	EU27
0.0904	0.2351	0.1638	0.2351	0.2351	cubic meter	natural gas, high pressure	regasification LNG from US	EU27

Table S16. Life cycle inventory for the natural gas supply distribution within EU countries across scenarios compiled from the ecoinvent database [S18], related to STAR Methods.

Across scenarios	Unit	Product	Activity	Location
		**Reference product**		
1.000	Cubic meter	natural gas distribution within EU27	natural gas distribution within EU27	EU27
		**Technosphere flows**		
0.0002	kilowatt hour	electricity, medium voltage	market for electricity, medium voltage	PL
0.0001				HU
0.0001				AT
0.0001				CZ
0.0000				GR
0.0000				SK
0.0000				IE
0.0000				DK
0.0000				FI
0.0000				SE
0.0110	megajoule	heat, district or industrial, natural gas	market for heat, district or industrial, natural gas	Europe without Switzerland
0.0910		natural gas, burned in gas turbine	natural gas, burned in gas turbine	DE
0.0400				IT
0.0319				FR
0.0196				NL
0.0166				ES
0.0163				RoE
0.0080				BE
0.0079				RO
0.0049	cubic meter	natural gas, high pressure	natural gas, high pressure, domestic supply with seasonal storage	NL
0.0186			petroleum and gas production, onshore	RO
0.0110			natural gas, high pressure, domestic supply with seasonal storage	DE
0.0065			petroleum and gas production, offshore	RO
0.0152	kilometer	pipeline, natural gas, high pressure distribution network	pipeline construction, natural gas, high pressure distribution network	Europe without Switzerland

Table S17. Estimated yearly per capita carbon budgets from the beginning of 2020 based on IPCC AR6 [S2], related to Figure 2. The global cumulative budget of CO_2 emissions provided by the IPCC AR6 was downscaled to the cumulative EU population over the same period (2020-2100) to obtain a constant per capita carbon budget**.** The remaining yearly kg of CO2 emission per capita varies with the temperature goal and likelihood of attaining it.

Temperature increase goal	Likelihood of limiting global warming to temperature goal				
	17%	33%	50%	67%	83%
1.5 °C	1,126	813	626	501	375
1.7 °C	1,815	1,314	1,064	876	688
2.0 °C	2,878	2,127	1,689	1,439	1,126

Table S18. Earth's carrying capacity threshold for EF impact assessment categories [S6], relate to STAR Methods. The designated 16 impact categories that form the EF framework of the European Commission [S3] updated version 3.1 [S21]. These global threshold values proposed in Sala et al. [S6] were derived from the Planetary Boundaries framework, which delineates the safe operating space for humanity with respect to various Earth system processes based on a comprehensive set of ecological and environmental metrics [S7, S8, S22]. Global per capita carrying capacity is scaled to the EU level following the egalitarian principle [S23].

EF category	Impact assessment method	Annual per capita carrying capacity[17]	EU's carrying capacity	Unit	Planetary Boundary classification
Acidification	EF 3.1	$1.45 \cdot 10^{2}$	$6.48 \cdot 10^{10}$	mol H^{+}-eq $year^{-1}$	
Climate change‡	IPCC 2021	$5.01 \cdot 10^{2}$‡	$2.24 \cdot 10^{11}$	kg CO_2 $year^{-1}$	
Ecotoxicity, freshwater	EF 3.1	$1.90 \cdot 10^{4}$	$8.49 \cdot 10^{12}$	CTUe $year^{-1}$	Climate action, water and terrestrial life protection
Eutrophication, freshwater	EF 3.1	$8.40 \cdot 10^{-1}$	$1.30 \cdot 10^{11}$	kg P-eq $year^{-1}$	
Eutrophication, marine	EF 3.1	$2.90 \cdot 10^{1}$	$3.96 \cdot 10^{12}$	kg N-eq $year^{-1}$	
Eutrophication, terrestrial	EF 3.1	$8.87 \cdot 10^{2}$	$3.96 \cdot 10^{11}$	mol N-eq $year^{-1}$	
Human toxicity, carcinogenic	EF 3.1	$1.39 \cdot 10^{-4}$	$6.21 \cdot 10^{4}$	CTUh $year^{-1}$	
Human toxicity, non-carcinogenic	EF 3.1	$5.93 \cdot 10^{-4}$	$2.65 \cdot 10^{5}$	CTUh $year^{-1}$	Human health
Ionising radiation	EF 3.1	$7.62 \cdot 10^{4}$	$3.41 \cdot 10^{13}$	kBq U235-eq $year^{-1}$	
Land use, soil erosion	LANCA v2.5 [S20]	$1.84 \cdot 10^{3}$	$8.22 \cdot 10^{11}$	kg soil loss $year^{-1}$	Climate action, water and terrestrial life protection
Ozone depletion	EF 3.1	$7.80 \cdot 10^{-2}$	$3.49 \cdot 10^{7}$	kg CFC-11-eq $year^{-1}$	
Particulate matter formation	EF 3.1	$7.47 \cdot 10^{-5}$	$3.34 \cdot 10^{4}$	disease incidence $year^{-1}$	Human health
Photochemical ozone formation	EF 3.1	$5.88 \cdot 10^{1}$	$2.63 \cdot 10^{10}$	kg NMVOC-eq $year^{-1}$	
Non-renewable energy resource use	EF 3.1	$3.24 \cdot 10^{4}$	224	EJ $year^{-1}$	Resource use
Mineral and metals resource use	EF 3.1	$3.18 \cdot 10^{-2}$	$1.42 \cdot 10^{7}$	kg Sb-eq $year^{-1}$	
Water use	EF 3.1	$2.63 \cdot 10^{4}$	$1.18 \cdot 10^{13}$	m^3 world-eq $year^{-1}$	Climate action, water and terrestrial life protection

‡ Carbon budget attaining 1.5 °C temperature increase goal with high probability (67%) [S2]**.**

Table S19. Overview of the recommended impact categories, impact assessment methods, and their quality levels [S24–S26], related to STAR Methods. Level I methods are considered recommended and satisfactory and appropriate for all LCA applications. Level II methods are but necessitate a clearer articulation of uncertainties and a more rigorous evaluation of their effects, alongside a call for research to improve aspects such as precision and flow coverage. Level III stands for methods that are recommended but to be applied with caution and indicates methods with substantial uncertainties or other deficiencies. The use of Level III methods demands careful application, and additional research, and should be accompanied by a presentation of results with and without these methods, as well as sensitivity analyses using alternatives to highlight differences in LCA interpretation.

Impact category	Impact assessment method	Quality level
Acidification	EF 3.1	II
Climate change	IPCC 2021	I
Ecotoxicity, freshwater	EF 3.1	II/III
Eutrophication, freshwater	EF 3.1	II
Eutrophication, marine	EF 3.1	II
Eutrophication, terrestrial	EF 3.1	II
Human toxicity, carcinogenic	EF 3.1	II/III
Human toxicity, non-carcinogenic	EF 3.1	II/III
Ionising radiation	EF 3.1	II
Land use, soil erosion	LANCA v2.5 [S20]	III
Ozone depletion	EF 3.1	I
Particulate matter formation	EF 3.1	I
Photochemical ozone formation	EF 3.1	II
Non-renewable energy resource use	EF 3.1	III
Mineral and metals resource use	EF 3.1	III
Water use	EF 3.1	III

Table S20. Life cycle inventory example for technosphere matrix sensitivity analysis [S27], related to STAR Methods. The provided example accounts for the equivalent switch of 1 bcm of natural gas- to coal-fired power plants in the EU. The equivalent replacement of one bcm of natural gas to coal in electricity production in power plants can be achieved by changing 4.48 TWh of the electricity production. In the life cycle inventory of electricity originally produced by natural gas, 4.48 TWh accounts for 0.79% (out of 569.4 TWh) that should be subtracted from the electricity production from the natural gas activity and added to the electricity production from coal.

Base	Coal-fired power plant increment	Unit	Product	Activity	Location
**Reference product**					
1.000	1.000	Kilowatt hour	Electricity from NG	Electricity production from NG	EU27
**Technosphere flows**					
0.4442	0.4353	kilowatt hour	electricity, high voltage	electricity production, NG	EU27
0.5558	0.5558			electricity production, NG, CHP	
0.0	0.0089			electricity production, coal	
0.0	0.0			electricity production, coal, CHP	
0.0	0.0			electricity production, nuclear	
0.0	0.0			electricity production, wind	
0.0	0.0			electricity production, photovoltaic	
0.0	0.0			electricity production, biomass, CHP	
0.0	0.0			electricity production, oil	
0.0	0.0			electricity production, oil, CHP	
0.0	0.0			electricity production, lignite	

Methods S1. Uncertainty analysis, related to STAR Methods

We considered uncertainties in the life cycle inventory (technosphere and biosphere data). These uncertainties were assessed based on uncertainty models (mostly normal and log-normal probability distributions) and parameters provided by the ecoinvent 3.9.1 database [S18]. From that, we generated 1000 data points using Monte Carlo sampling. Each sample entails a set of values of the technosphere and biosphere matrices, for which the LCIA can be carried out across scenarios using the Environmental Footprint (EF) v3.1 [S19], LANCA v2.5 [S20], IPCC 2021 [S2], and life-cycle CO_2 emission impact assessment methods.

Monte Carlo simulations

Table S4 – **Table S8** summarise the outcome of the LCA uncertainty analysis using the EF v3.1 [S19], LANCA v2.5 [S20], IPCC 2021 [S2], and life-cycle CO_2 impact assessment methods for the EU's natural gas supply and demand for energy use scenarios Base, REPowerEU, Coal, and Clean, respectively, as described in the main text. These tables provide essential statistics, including the baseline LCIA result, mean, standard deviation (Std), quartile coefficient of dispersion (QCD), minimum, maximum, and quartile values. The QCD measures the dispersion of stochastic data more robustly with respect to outliers [S28] and can be defined as

$$QCD = \frac{Q_3 - Q_1}{Q_3 + Q_1},$$

in which Q_1 and Q_3 stand for the first and third quartiles, respectively, i.e. the values that split off the lowest 25% and 75% of the data from the rest. These results enable a more robust comparison of the environmental performance of the analysed scenarios of natural gas supply and demand in the EU.

We highlight that the discrepancy between the baseline, i.e. the LCIA results for the nominal values of the technosphere and biosphere, and the mean or median of the Monte Carlo outcome, is due to non-centred uncertainty models considered in ecoinvent 3.9.1, e.g. log-normal distributions (**Figure S5**) and their propagation through the whole life cycle of our system. We also note that the QCD values for freshwater ecotoxicity and human toxicity (carcinogenic and noncarcinogenic) are extremely high (> 100%); therefore, their baseline results should be used carefully.

Scenario Comparison

To determine whether the difference between the mean of the 1000 scenarios generated via Monte Carlo simulation in the Base scenario and the analogue in the alternative cases is statistically significant, we use the p-value of a paired sample t-test [S4]. For that, we consider each Monte Carlo simulation across scenarios to be correlated, as they resulted from technosphere and biosphere matrices generated from the same random numbers (fixed seed). **Figure S8** presents the p-value results of the paired sample t-test, where a value below 0.05 allows rejecting the null hypothesis of equal means, indicating that the two scenarios being compared are statistically different for the impact metric under study.

In order to compare the outcomes of the Monte Carlo simulation for each scenario, we calculate the probability that the alternative scenario (REPowerEU, Coal, and Clean) has a greater life cycle impact than the Base scenario. For each Monte Carlo sample, all scenarios share the same stochastically generated technosphere and biosphere matrices, so the LCIA results can be compared considering a one-to-one relation. Therefore, the probability of the outcome A (an alternative scenario) being greater than B (Base scenario), considering their uncertainty, is given by the discernibility factor of dependent datasets [S4,S29], such that:

$$P(A > B) = \frac{1}{\mathrm{n}} \sum_{\mathrm{i}=1}^{\mathrm{n}} \Theta(\boldsymbol{a}_i - \boldsymbol{b}_i),$$

where Θ is the Heaviside step function (returns 1 if its argument is positive and 0 otherwise), $\boldsymbol{a}, \boldsymbol{b} \in \mathbb{R}^n$ are the Monte Carlo simulation outcomes for the alternative and Base scenarios, respectively, and n is the number of simulations.

Methods S2. Life cycle assessment, related to STAR Methods

Here we further describe the methods used to evaluate the life-cycle environmental sustainability across scenarios, the absolute environmental impacts based on carbon budgets for temperature increase targets and the Earth's carrying capacity as well as quality levels and sensitivity analysis.

Computation structure of life cycle assessments

Following the standards proposed in the ISO 14040 and 14044 [S30,S31] and considering the functional unit defined in **Table S10** and inventories across scenarios defined in **Table S11** – **Table S16** on top of the ecoinvent v3.9.1 database [S32], we compute our systems' life cycle impacts using the open-source Brightwat2 software [S33]. In order to carry out an LCIA calculation, let us first define the technosphere matrix ($A \in \mathbb{R}^{p \times p}$) such that each element $a_{i,j}$ consists of the mass or energy from or to the economy (e.g., amount of natural gas required to produce 1 kWh of electricity) for all p processes [S27]. Similarly, the biosphere matrix ($B \in \mathbb{R}^{e \times p}$) is all the e mass and energy flows from/to the environment associated with all p processes. Those matrices allow us to compute the inventory vector (g), which comprises the life-cycle exchanges to/from the environment, such that

$$g = B\,A^{-1}\,f,$$

in which f is the final demand vector defined by our functional unit [S27]. Finally, we can use a matrix of characterisation factors ($Q \in \mathbb{R}^{k \times e}$) that penalizes all e exchange in the inventory vector g to calculate their associated environmental impacts across all k impact categories [S27] (e.g., the life-cycle emission of 1 kg of CO_2 to the air has a 1 kg CO_2-eq impact on climate change accordingly to the IPCC 2021, 100-year averaged GWP method [S2]).

Impact assessment methods

In this section, we describe all impact assessment methods employed throughout our work, i.e., the IPCC 2021 [S2], Environmental Footprint (EF) v3.1 [S19], and LANCA v2.5 [S20] methods, in terms of underlying models, assumptions, units, and importance. More specifically, we employ the 100-year and 20-year GWP methods published by the IPCC to account for climate change. Additionally, we consider 15 impact categories included in the Environmental Footprint v3.1 methods recommended by the European Commission [S3,S21] (i.e., acidification; freshwater ecotoxicity; energy resource non-renewable consumption; freshwater, marine, and terrestrial eutrophication; carcinogenic and noncarcinogenic human toxicity; ionising radiation; land use; material resource use; ozone depletion; particulate matter formation; photochemical oxidant formation; and water use). Finally, we also consider the soil erosion potential from the LANCA v2.5 method.

Climate change: The 100-year and 20-year GWP metrics as outlined by the IPCC AR6 report to assess climate change impacts measure the relative heat trapped by anthropogenic GHG emissions (e.g., CO_2, CH_4, N_2O, fluorinated gases, etc.) over the specified time horizons, expressed in units of kilogram of CO_2 equivalents (kg CO_2-eq). The 100-year GWP is widely used for policy-making due to its longer-term perspective, while the 20-year GWP highlights near-term climate impacts, emphasising the urgency of immediate action.

Environmental Footprint (EF) v3.1: This sustainability assessment method, recommended by the European Commission [S3,S21], includes additional 15 impact categories as follow:

- **Acidification [S34]:** measures the potential impacts on terrestrial ecosystems due to the accumulated exceedance of acidifying substances (e.g., NO_x, NH_3 and SO_x). Computed in the equivalent mol of hydrogen ions released (mol H^+-eq), which leads to soil and water acidification.
- **Ecotoxicity, freshwater [S35]:** assesses the impact of toxic substances on freshwater ecosystems. Employing the USEtox v2.1 model, it computes the comparative toxic unit for aquatic ecotoxicity impacts (CTUe), which expresses the estimated potentially affected fraction of species per cubic meter of freshwater compartment per day.
- **Energy resource, non-renewable [S36]:** quantifies the depletion of non-renewable energy resources based on abiotic depletion potential (ADP). The latter is calculated as the ratio between (i) the fossil energy consumption over the ultimate reserve of fossil fuels and (ii) the extraction rate of the reference source (antimony) and the ultimate reserve of the reference resource. The ADP weights the consumption of the non-renewable energy resource and the overall impact is in MJ.

- **Eutrophication, freshwater [S37]:** quantifies phosphorus-based nutrient emissions to water and soil that reaches freshwater ecosystems. The potential excessive plant growth and ecosystem imbalance impact is calculated in kilogram of phosphorus equivalents (kg P-eq).
- **Eutrophication, marine [S37]:** quantifies nitrogen-based nutrient emissions to water that reaches marine ecosystems. The potential excessive plant growth and ecosystem imbalance impact is calculated in kilogram of nitrogen equivalents (kg N-eq).
- **Eutrophication, terrestrial [S34]:** like acidification, it assesses nutrient enrichment in terrestrial systems based on accumulated exceedance of nitrogen compounds (NH3, NOx, and nitrate), which can lead to excessive plant growth and ecosystem imbalance. It is calculated in mol of nitrogen equivalent (mol N-eq).
- **Human toxicity (carcinogenic, noncarcinogenic) [S37]:** evaluates health risks from ingestion and inhalation exposure to hazardous substances. Based on USEtox v2.1 model, it computes the comparative toxic unit for human toxicity impacts (CTUh), which entails the estimated increase in in morbidity (the number of carcinogenic and noncarcinogenic disease cases) in the total human population.
- **Ionising radiation [S38]:** quantifies potential human health damages based on the concept of disability adjusted life years (DALY) related to anthropogenic radioactive material releases to the environment, expressed in thousands of becquerel (radioactive decay per second) of uranium 235 isotope equivalent (kBq U235-eq).
- **Land use, soil quality [S39]:** aggregates linearly in a dimensionless soil quality index the impacts of land occupation and transformation on erosion resistance, mechanical filtration, groundwater regeneration, and biotic production from the LANCA v2.5 model [S20].
- **Material resource: metals and minerals [S36]:** similar to energy resources non-renewable, it quantifies the depletion of material (mineral and metals) resources based on ADP. Here, the latter is calculated as the ratio between (i) the extraction rate of minerals and metals over their ultimate reserve and (ii) the extraction rate of the reference source (antimony) and the ultimate reserve of the reference resource. The ADP weights the consumption of materials and the overall impact is in kilogram of antimony equivalent (kg Sb-eq).
- **Ozone depletion [S40]:** computes the destruction potential of the stratospheric ozone layer, which is responsible for reducing the amount of UV radiation that reaches the ground, from emissions to air over a 100-year time horizon. This method normalises the impacts of several emissions (mainly halogenated gases) based on those from trichlorofluoromethane (CFC-11) and therefore is calculated in kg CFC-11-eq.
- **Particulate matter formation [S41]:** estimates the impacts on human health attributed to exposure to fine particulate matter or gases that are precursors of particulate matter by oxidation (i.e., NO_x, SO_2, and NH_3). The exposure model takes into account the amount of particulate matter intake per pollutant emitted depending on the emission compartment (outdoor urban, outdoor rural, indoor high concentrations, or indoor low concentration) and emission source type (ground level, low stack, high stack, or very high stack) to compute the metric in disease incidences.
- **Photochemical ozone formation:** assesses the ground-level ozone formation potentials that cause smog and may lead to human health issues. Ozone and other reactive oxygen compounds are formed from the oxidation of contaminants close to the ground (mainly volatile organic compounds) in the presence of light. This method normalises the impact of several emissions to the air based on those from non-methane volatile organic compounds (NMVOC) and therefore is calculated in kg NMVOC-eq.
- **Water use [S42]:** quantifies impacts of water consumption on local scarcity of freshwater resources at different locations in m^3 of world water deprived. This metric is weighted by the available water remaining per area in a watershed after the demand of humans and aquatic ecosystems has been met.

Land use, soil erosion: The Land Use Indicator Value Calculation in Life Cycle Assessment (LANCA) method v2.5 [S20] is employed to provide a detailed evaluation of impacts on soil erosion due to land occupation and transformation, in kilograms of soil loss.

Methods S3. Methodological assumptions and limitations, related to STAR Methods

Here we list the main assumptions and limitations of the assessed natural gas supply and demand scenarios and LCIA results.

- We assumed cradle-to-gate system boundaries to our LCA, ensuring a comprehensive assessment of the environmental impacts of energy production (heat and electricity) from the energy resource extraction up to the electricity and heat production. That allows accounting for all emissions and resource use throughout the process life cycle. This approach is robust to understanding the full environmental footprint of energy production within the system but lacks addressing post-gate activities such as distribution, use, and end-of-life stages.
- We considered our LCA system temporal scope to be on an annual basis. This assumption overlooks seasonal variations, such as the increased demand for natural gas during winter months for individual heating purposes. By not accounting for these dynamics, the LCA may not fully capture the temporal distribution of emissions and resource use, potentially leading to a less accurate representation of the environmental impact. Such an average annual model may mask peak load impacts on infrastructure and emissions, as well as the potential benefits of demand-side management and storage solutions designed to address variability in supply and demand throughout the year.
- The scenarios presented here are simplified snapshots of the natural gas crisis and the EU's policy actions to overcome it. This static view of the European natural gas supply and demand scenarios might not accurately reflect the dynamic and hard-to-predict nature of such systems (e.g., new policy decisions, geopolitical change, or new market responses). In addition, the scenarios do not incorporate the uncertainty that is inherent in the foreground model, such as the variability in production efficiency, diverse technology performance, or operational practices, where an averaged approach based on the current situation was employed.
- The current environmental impact analysis overlooks the geographical implications of diversifying natural gas suppliers and the array of demand-side measures. Different countries may experience more pronounced effects based on their energy profiles (e.g., higher reliance on natural gas) and policy decisions (e.g., the delayed phase-out of nuclear and coal-fired power plants). Additionally, the scenarios do not consider the uneven impact of household heating savings, which could significantly affect energy demand and emission profiles regionally due to weather and economic differences among European countries, for instance.
- The lack of a regionalized life cycle inventory and uncertainties around the safe operating space and damage factors pose significant limitations. Without regionalized data, the scenarios cannot accurately reflect the localized environmental impacts of the natural gas supply chain and its energy demand (electricity and heat) production. The lack of uncertain information on the safe operating spaces for different Earth's processes and damage factors hampers the statistically significant interpretation of the environmental impact results both for scenario comparison and absolute results.
- The analysis falls short in providing detailed insights into the utilization of natural gas for energy purposes at the plant level. This lack of granularity hinders the ability to fully model the demand-side policy actions to higher precision, e.g., delaying the phase-out of specific nuclear or coal-fired power plants. Also, the lack of plant-level information prohibits the consideration of accurate efficiency, for which an averaged value was employed.
- The current analysis disregards the impacts in externalities associated with restructuring the natural gas supply chain and its energy-related demand in the EU, e.g., rise in energy prices, increased energy demand for LNG production externally from the EU, and global economic disruptions. The present assessment focuses on the directly affected countries by the natural gas supply disruption, i.e. EU countries.

Supplemental references

[S1] Pedersen, T.T., Gøtske, E.K., Dvorak, A., Andresen, G.B., and Victoria, M. (2022). Long-term implications of reduced gas imports on the decarbonization of the European energy system. Joule 6, 1566–1580. https://doi.org/10.1016/j.joule.2022.06.023.

[S2] IPCC (2021). Climate Change 2021: The Physical Science Basis. Intergovernmental Panel on Climate Change (IPCC). https://www.ipcc.ch/report/ar6/wg1/

[S3] European Commission (2021). Commission Recommendation of 16.12.2021 on the use of the Environmental Footprint methods to measure and communicate the life cycle environmental performance of products and organisations. https://eur-lex.europa.eu/legal-content/EN/TXT/?uri=CELEX:32021H2279

[S4] Heijungs, R. (2021). Selecting the best product alternative in a sea of uncertainty. Int J Life Cycle Assess 26, 616–632. https://doi.org/10.1007/s11367-020-01851-4.

[S5] Dao, H., Peduzzi, P., and Friot, D. (2018). National environmental limits and footprints based on the Planetary Boundaries framework: The case of Switzerland. Global Environmental Change 52, 49–57. https://doi.org/10.1016/j.gloenvcha.2018.06.005.

[S6] Sala, S., Crenna, E., Secchi, M., and Sanyé-Mengual, E. (2020). Environmental sustainability of European production and consumption assessed against planetary boundaries. Journal of Environmental Management 269, 110686. https://doi.org/10.1016/j.jenvman.2020.110686.

[S7] Rockström, J., Steffen, W., Noone, K., Persson, Å., Chapin, F.S., Lambin, E., Lenton, T.M., Scheffer, M., Folke, C., Schellnhuber, H.J., et al. (2009). Planetary Boundaries: Exploring the Safe Operating Space for Humanity. Ecology and Society 14. http://www.jstor.org/stable/26268316

[S8] Steffen, W., Richardson, K., Rockström, J., Cornell, S.E., Fetzer, I., Bennett, E.M., Biggs, R., Carpenter, S.R., De Vries, W., De Wit, C.A., et al. (2015). Planetary boundaries: Guiding human development on a changing planet. Science 347, 1259855. https://doi.org/10.1126/science.1259855.

[S9] O'Neill, D.W., Fanning, A.L., Lamb, W.F., and Steinberger, J.K. (2018). A good life for all within planetary boundaries. Nat Sustain 1, 88–95. https://doi.org/10.1038/s41893-018-0021-4.

[S10] Bjørn, A., Sim, S., King, H., Patouillard, L., Margni, M., Hauschild, M.Z., and Ryberg, M. (2020). Life cycle assessment applying planetary and regional boundaries to the process level: a model case study. Int J Life Cycle Assess 25, 2241–2254. https://doi.org/10.1007/s11367-020-01823-8.

[S11] Guinée, J.B., de Koning, A., and Heijungs, R. (2022). Life cycle assessment-based Absolute Environmental Sustainability Assessment is also relative. Journal of Industrial Ecology 26, 673–682. https://doi.org/10.1111/jiec.13260.

[S12] European Commission (2022). REPowerEU Plan. https://eur-lex.europa.eu/legal-content/EN/TXT/?uri=COM:2022:230:FIN

[S13] European Commission (2022). Implementing the REPowerEU action plan: Investment needs, hydrogen accelerator and achieving the bio-methane targets. https://eur-lex.europa.eu/legal-content/EN/TXT/?uri=SWD:2022:230:FIN

[S14] bp (2022). Statistical Review of World Energy 2022. https://www.bp.com/en/global/corporate/energy-economics/webcast-and-on-demand.html

[S15] Zachmann, G., McWilliams, B., Keliauskaite, U., and Sgaravatti, G. (2024). European natural gas imports. https://www.bruegel.org/dataset/european-natural-gas-imports.

[S16] Eurostat (2024). Complete energy balances. https://doi.org/10.2908/NRG_BAL_C.

[S17] IEA (2023). Europe's energy crisis: What factors drove the record fall in natural gas demand in 2022? IEA. https://www.iea.org/commentaries/europe-s-energy-crisis-what-factors-drove-the-record-fall-in-natural-gas-demand-in-2022.

[S18] Wernet, G., Bauer, C., Steubing, B., Reinhard, J., Moreno-Ruiz, E., and Weidema, B. (2016). The ecoinvent database version 3 (part I): overview and methodology. Int J Life Cycle Assess 21, 1218–1230. https://doi.org/10.1007/s11367-016-1087-8.

[S19] Sala, S., Biganzoli, F., Mengual, E.S., and Saouter, E. (2022). Toxicity impacts in the environmental footprint method: calculation principles. Int J Life Cycle Assess 27, 587–602. https://doi.org/10.1007/s11367-022-02033-0.

[S20] Horn, R., and Maier, S. (2018). LANCA®-characterization factors for life cycle impact assessment, version 2.5. Fraunhofer: Stuttgart, Germany.

[S21] Andreasi Bassi, S., Biganzoli, F., Ferrara, N., Amadei, A., Valente, A., Sala, S. and Ardente, F. (2023). Updated characterisation and normalisation factors for the environmental footprint 3.1 method. Publications Office of the European Union, Luxembourg. https://dx.doi.org/10.2760/798894

[S22] Richardson, K., Steffen, W., Lucht, W., Bendtsen, J., Cornell, S.E., Donges, J.F., Drüke, M., Fetzer, I., Bala, G., von Bloh, W., et al. (2023). Earth beyond six of nine planetary boundaries. Science Advances 9, eadh2458. https://doi.org/10.1126/sciadv.adh2458.

[S23] Ryberg, M.W., Andersen, M.M., Owsianiak, M., and Hauschild, M.Z. (2020). Downscaling the planetary boundaries in absolute environmental sustainability assessments – A review. Journal of Cleaner Production 276, 123287. https://doi.org/10.1016/j.jclepro.2020.123287.

[S24] Michael, H., Mark, G., Jerome, G., Reinout, H., Mark, H., Olivier, J., Manuele, M., and An, D.S. (2011). Recommendations for Life Cycle Impact Assessment in the European context - based on existing environmental impact assessment models and factors. International Reference Life Cycle Data System - ILCD handbook.

[S25] Fazio, S., Castellani, V., Sala, S., Schau, E., Secchi, M., Zampori, L. and Diaconu, E. (2018). Supporting information to the characterisation factors of recommended EF Life Cycle Impact Assessment methods: New methods and differences with ILCD. EUR 28888 EN, Publications Office of the European Union, Luxembourg. https://dx.doi.org/10.2760/671368

[S26] Fazio, S., Biganzoli, F., De Laurentiis, V., Zampori, L., Sala, S. and Diaconu, E. (2018). Supporting information to the characterisation factors of recommended EF Life Cycle Impact Assessment methods, EUR 29600 EN, Publications Office of the European Union, Luxembourg. https://dx.doi.org/10.2760/002447

[S27] Heijungs, R., and Suh, S. (2002). The Computational Structure of Life Cycle Assessment (Springer Netherlands) https://doi.org/10.1007/978-94-015-9900-9.

[S28] Botta-Dukát, Z. (2023). Quartile coefficient of variation is more robust than CV for traits calculated as a ratio. Sci Rep 13, 4671. https://doi.org/10.1038/s41598-023-31711-8.

[S29] Heijungs, R., and Kleijn, R. (2001). Numerical approaches towards life cycle interpretation five examples. Int J LCA 6, 141–148. https://doi.org/10.1007/BF02978732.

[S30] ISO (2006). ISO 14040:2006 — Environmental management — Life cycle assessment — Principles and framework.

[S31] ISO (2006). ISO 14044:2006 — Environmental management — Life cycle assessment — Requirements and guidelines.

[S32] Wernet, G., Bauer, C., Steubing, B., Reinhard, J., Moreno-Ruiz, E., and Weidema, B. (2016). The ecoinvent database version 3 (part I): overview and methodology. Int. J. Life Cycle Assess. 21, 1218–1230. https://doi.org/10.1007/s11367-016-1087-8.

[S33] Mutel, C. (2017). Brightway: An open source framework for Life Cycle Assessment. JOSS 2, 236. https://doi.org/10.21105/joss.00236.

[S34] Seppälä, J., Posch, M., Johansson, M., and Hettelingh, J.-P. (2006). Country-dependent Characterisation Factors for Acidification and Terrestrial Eutrophication Based on Accumulated Exceedance as an Impact Category Indicator (14 pp). Int J Life Cycle Assessment 11, 403–416. https://doi.org/10.1065/lca2005.06.215.

[S35] Saouter, E., Biganzoli, F., Ceriani, L., Versteeg, D., Crenna, E., Zampori, L., Sala, S., and Pant, R. (2019). Environmental Footprint: Update of Life Cycle Impact Assessment methods – Ecotoxicity freshwater, human toxicity cancer, and non-cancer https://doi.org/10.2760/300987.

[S36] van Oers, L., de Koning, A., Guinée, J.B., and Huppes, G. (2002). Abiotic resource depletion in LCA - improving characterisation factors fro abiotic depletion as recommended in the new Dutch LCA Handbook Public Works and Water Management, the Netherlands, Delft.

[S37] Goedkoop, M., Heijungs, R., Huijbregts, M., De Schryver, A., Struijs, J., and van Zelm, R. (2013). ReCiPe 2008 A life cycle impact assessment method which comprises harmonised category indicators at the midpoint and the endpoint level. Report I: Characterisation factors.

[S38] Frischknecht, R., Braunschweig, A., Hofstetter, P., and Suter, P. (2000). Human health damages due to ionising radiation in life cycle impact assessment. Environmental Impact Assessment Review 20, 159–189. https://doi.org/10.1016/S0195-9255(99)00042-6.

[S39] De Laurentiis, V., Secchi, M., Bos, U., Horn, R., Laurent, A., and Sala, S. (2019). Soil quality index: Exploring options for a comprehensive assessment of land use impacts in LCA. Journal of Cleaner Production 215, 63–74. https://doi.org/10.1016/j.jclepro.2018.12.238.

[S40] World Meteorological Organization (2015). Scientific assessment of Ozone depletion: 2014. Pursuant to Article 6 of the Montreal Protocol on substances that deplete the ozone layer. World Meteorological Organization (WMO).

[S41] Fantke, P., Evans, J.R., Hodas, N., Apte, J.S., Jantunen, M.J., Jolliet, O., and McKone, T.E. (2016). Health impacts of fine particulate matter. In Global guidance for life cycle impact assessment indicators (SETAC), pp. 76–99.

[S42] Boulay, A.-M., Bare, J., Benini, L., Berger, M., Lathuillière, M.J., Manzardo, A., Margni, M., Motoshita, M., Núñez, M., Pastor, A.V., et al. (2018). The WULCA consensus characterization model for water scarcity footprints: assessing impacts of water consumption based on available water remaining (AWARE). Int J Life Cycle Assess 23, 368–378. https://doi.org/10.1007/s11367-017-1333-8.
